# Supplementary material for: Transient microglial absence assists postmigratory cortical neurons in proper differentiation
Source: Nat Commun. 2020 Apr 2;11:1631. doi: 10.1038/s41467-020-15409-3 (PMC7118101; doi:10.1038/s41467-020-15409-3)
Supplement: Supplementary file 1 — Supplementary Information [file 41467_2020_15409_MOESM1_ESM.pdf]

Supplementary Information

Transient microglial absence assists postmigratory cortical neurons in proper differentiation

Hattori et al.

Supplementary Figure 1

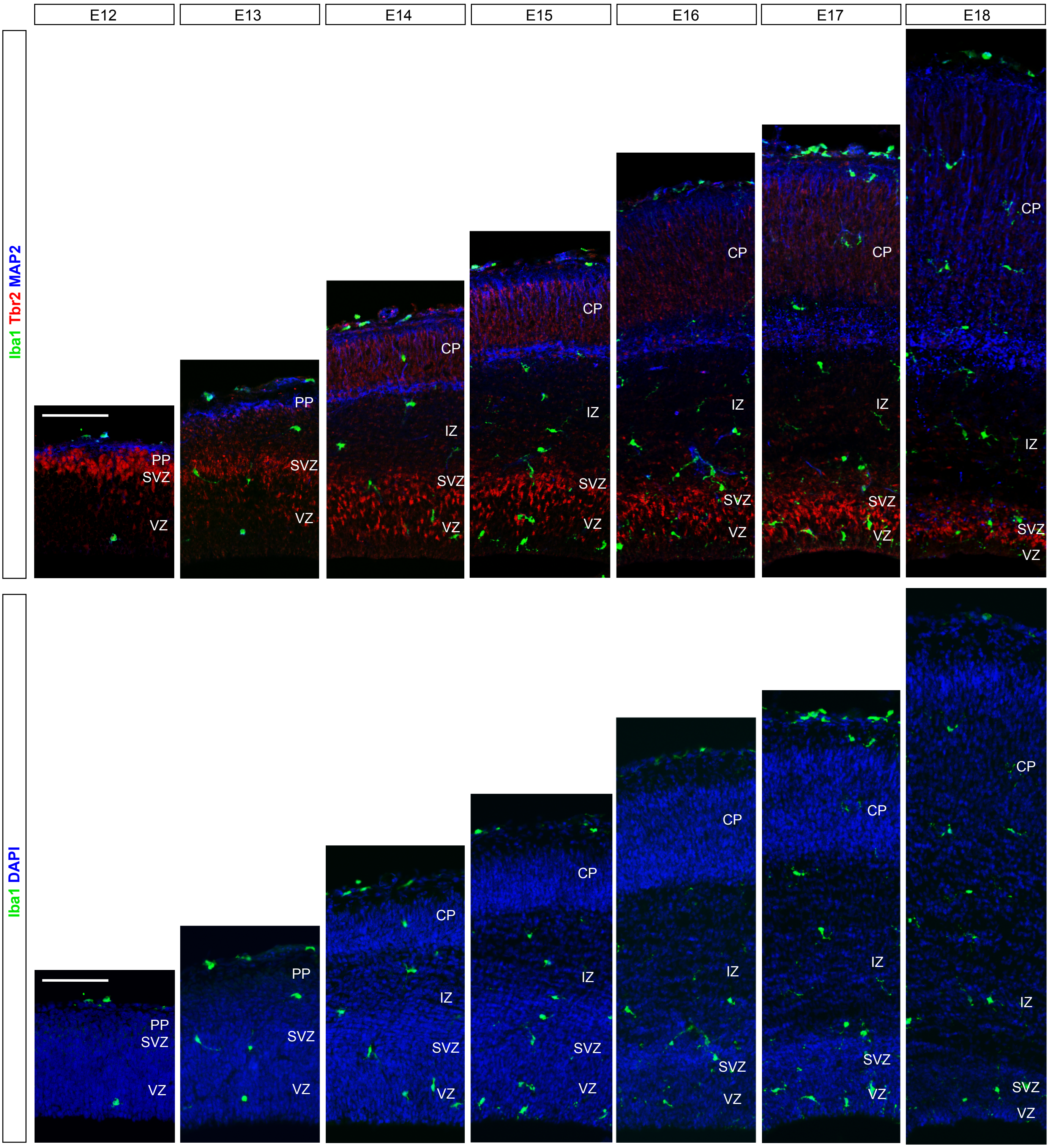

Supplementary Figure 1 Microglia are transiently absent from the CP in mouse cerebral walls from E15 to E16
Immunohistochemistry to detect Iba1 (microglia and perivascular macrophages), Tbr2 (intermediate progenitors and young neurons positioned in the SVZ), MAP2 (neurons) and DAPI in the developing cortex of E12–18 ICR mice. Scale bar, 100 µm.

Supplementary Figure 2

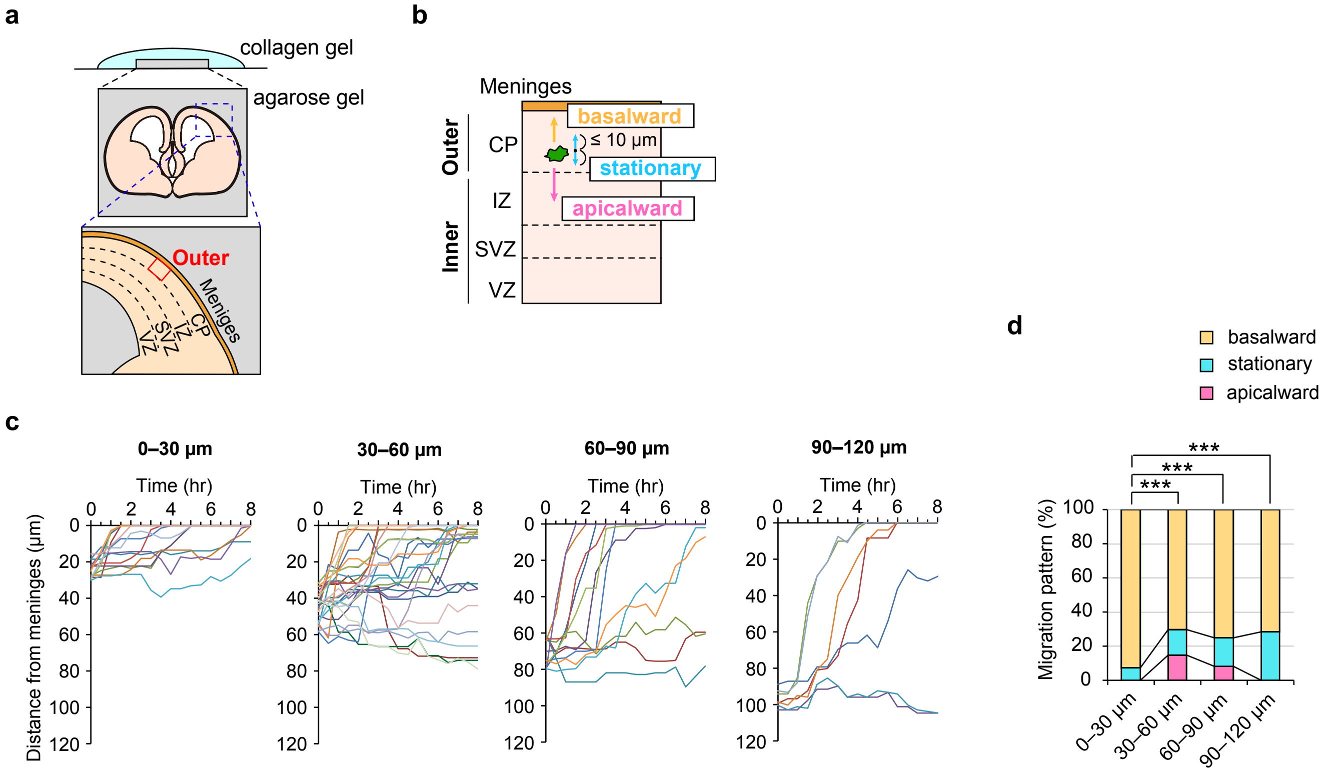

Supplementary Figure 2 Live-imaging of microglia in cerebral wall slices of CX3CR1-GFP mice
(**a**) Schematic of cultured cortical slices with intact meninges used for live imaging. (**b**) Microglia were categorized into three groups depending on their migration pattern over 8 hr: apicalward (> 10 µm towards the apical/ventricular surface), basalward (> 10 µm towards the meninges), or stationary (≤ 10 µm displacement). (**c**) The trajectories of the microglia, which were categorized into 4 groups by their position at time 0 (cells 0–30 µm, 30–60 µm, 60–90 µm and 90–120 µm deep relative to the meninges). (**d**) A comparison of microglial translocation patterns. The cells originally positioned near the meninges (0–30 µm deep relative to the meninges) moved basalward more easily than the cells that were positioned in more distant regions (two-sided Pearson's chi-squared test; n = 14, 27, 12, 7 cells (left to right); $P = 3.4 \times 10^{-5}$ (30–60 µm), $P = 8.9 \times 10^{-4}$ (60–90 µm), $P = 1.6 \times 10^{-4}$ (90–120 µm). Source data are provided as a Source Data file.

Supplementary Figure 3

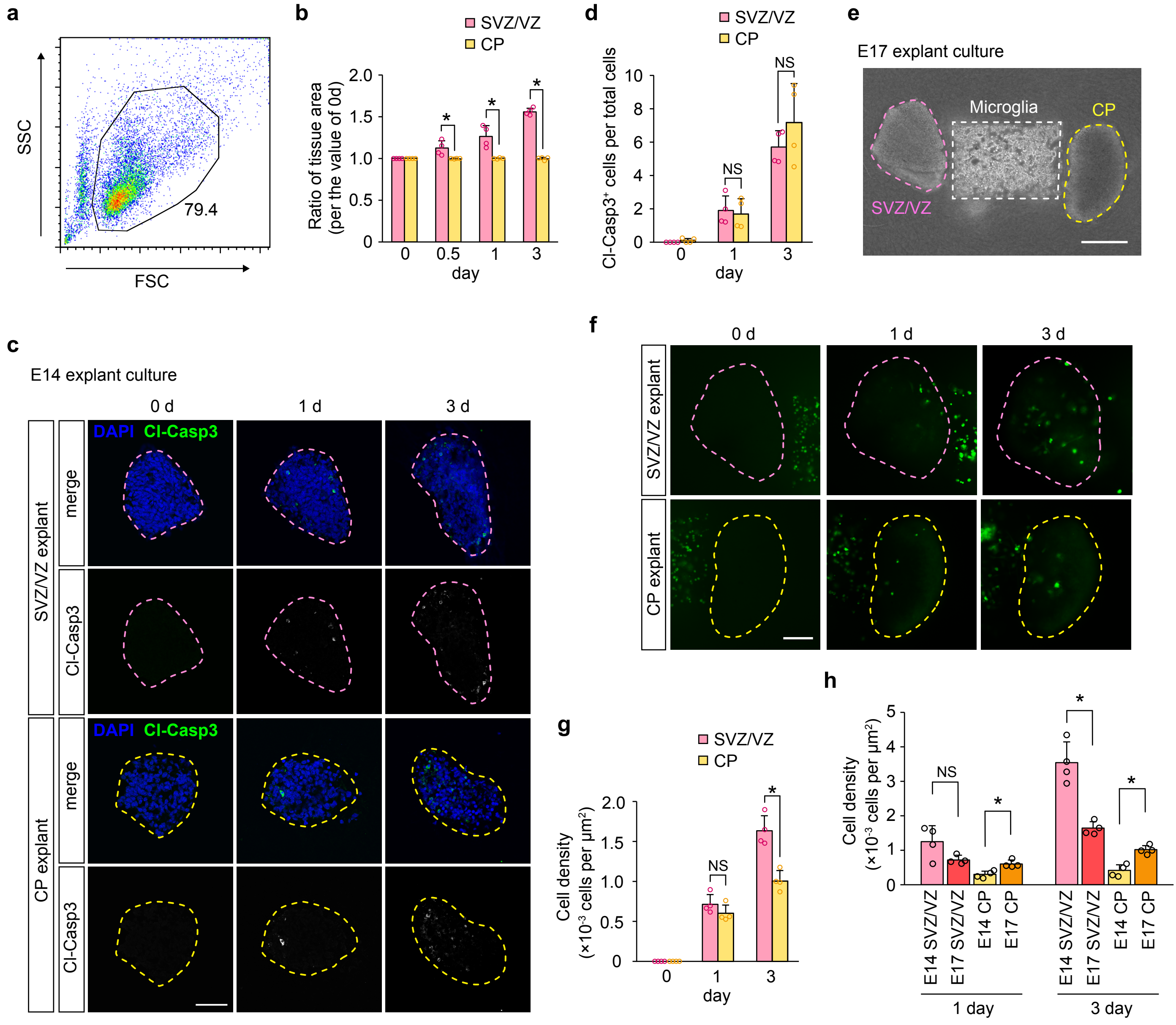

Supplementary Figure 3 The explant culture of cerebral walls together with isolated CX3CR1-GFP⁺ microglia
(**a**) The plot shows a FSC/SSC gating strategy of cerebral wall cells collected from CX3CR1-GFP E14 mice for CX3CR1-GFP⁺ cell isolation. (**b**) A graph depicting the ratio of tissue area of the CP and SVZ/VZ explants at 0.5, 1, and 3 d compared to that at 0 d, indicating that the SVZ/VZ explants grew more rapidly than the CP explants (two-sided Mann-Whitney U test; n = 4 independent cultures; P = 0.029 for 0.5 d, P = 0.029 for 1 d, P = 0.029 for 3 d). (**c**) Immunostaining of the explants cultured for 0, 1 or 3 d for Cl-Casp-3⁺ cells (cells undergoing apoptosis; green) and DAPI (blue). Scale bar, 50 μm. (**d**) A comparison of the number of Cl-Casp-3⁺ cells per total (DAPI⁺) cells. We did not detect a significant difference between that in the SVZ/VZ and CP explants, indicating that both explants were healthily cultured for 3 d (two-sided Mann-Whitney U test; n = 4 independent cultures; P = 0.686 for 1 d, P = 0.686 for 3 d). (**e**) A bright-field image of microglia (white square space) isolated from E14 CX3CR1-GFP mice adjacently cocultured with the SVZ/VZ and CP explants derived from E17 ICR mice. Scale bar, 100 μm. (**f**) Representative data of the monitoring the accumulation of CX3CR1-GFP⁺ cells in the SVZ/VZ (pink) and CP (yellow) explants. Scale bar, 50 μm. (**g**) A comparison of the density of microglia in the SVZ/VZ and CP explants 1 d or 3 d after the start of culture (two-sided Mann-Whitney U test; n = 4 independent cultures; P = 0.200 for 1 d, P = 0.029 for 3 d). (**h**) A graph depicting the density of microglia obtained from E14 brains in the SVZ/VZ and CP explants, which were derived from E14 and E17 mouse cerebral walls (two-sided Mann-Whitney U test; n = 4 independent cultures; P = 0.343, 0.029, 0.029 and 0.029 [left to right]). Data are presented as the mean values ± S.D. Source data are provided as a Source Data file.

Supplementary Figure 4

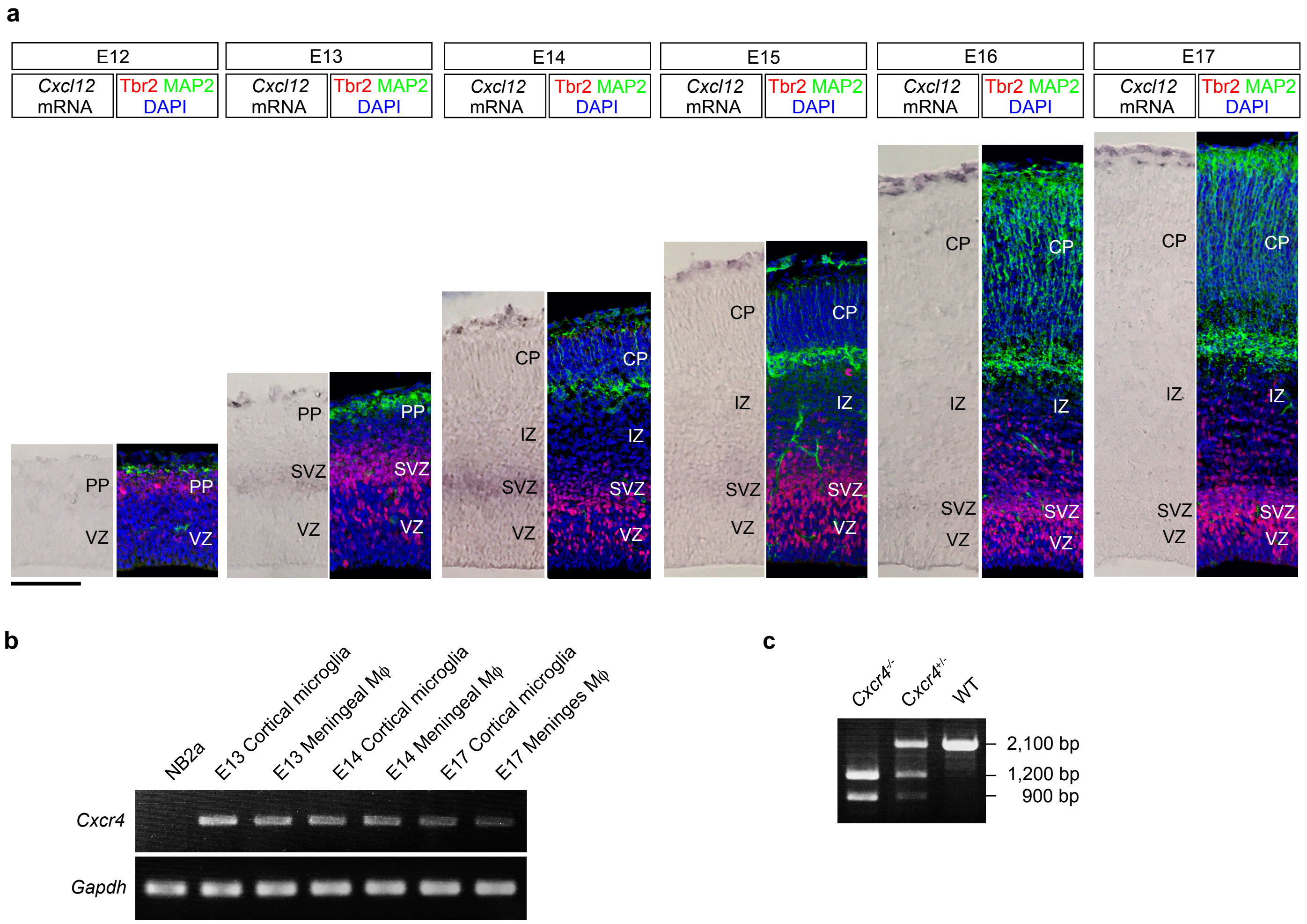

Supplementary Figure 4 The expression of Cxcl12 and Cxcr4 in the embryonic brain

(**a**) *In situ* hybridization for *Cxcl12* mRNA expression (left) and immunostaining for Tbr2/MAP2/DAPI (right) in E12–E17 cerebral walls. Scale bar, 100 µm. (**b**) The mRNA levels of *Cxcr4* (upper panel) and *Gapdh* (lower panel) were detected by RT-PCR in cDNA samples obtained from CX3CR1^{+} cells derived from the pallium or meninges of E13, E14, and E17 CX3CR1-GFP mice and NB2a cells (mouse neuroblastoma cell line). Mφ, perivascular macrophages. (**c**) Genotyping for *Cxcr4* from genome DNA samples derived from *Cxcr4*$^{-/-}$, *Cxcr4*$^{+/-}$ and WT mice (see methods for detailed information). Source data are provided as Source Data file.

Supplementary Figure 5

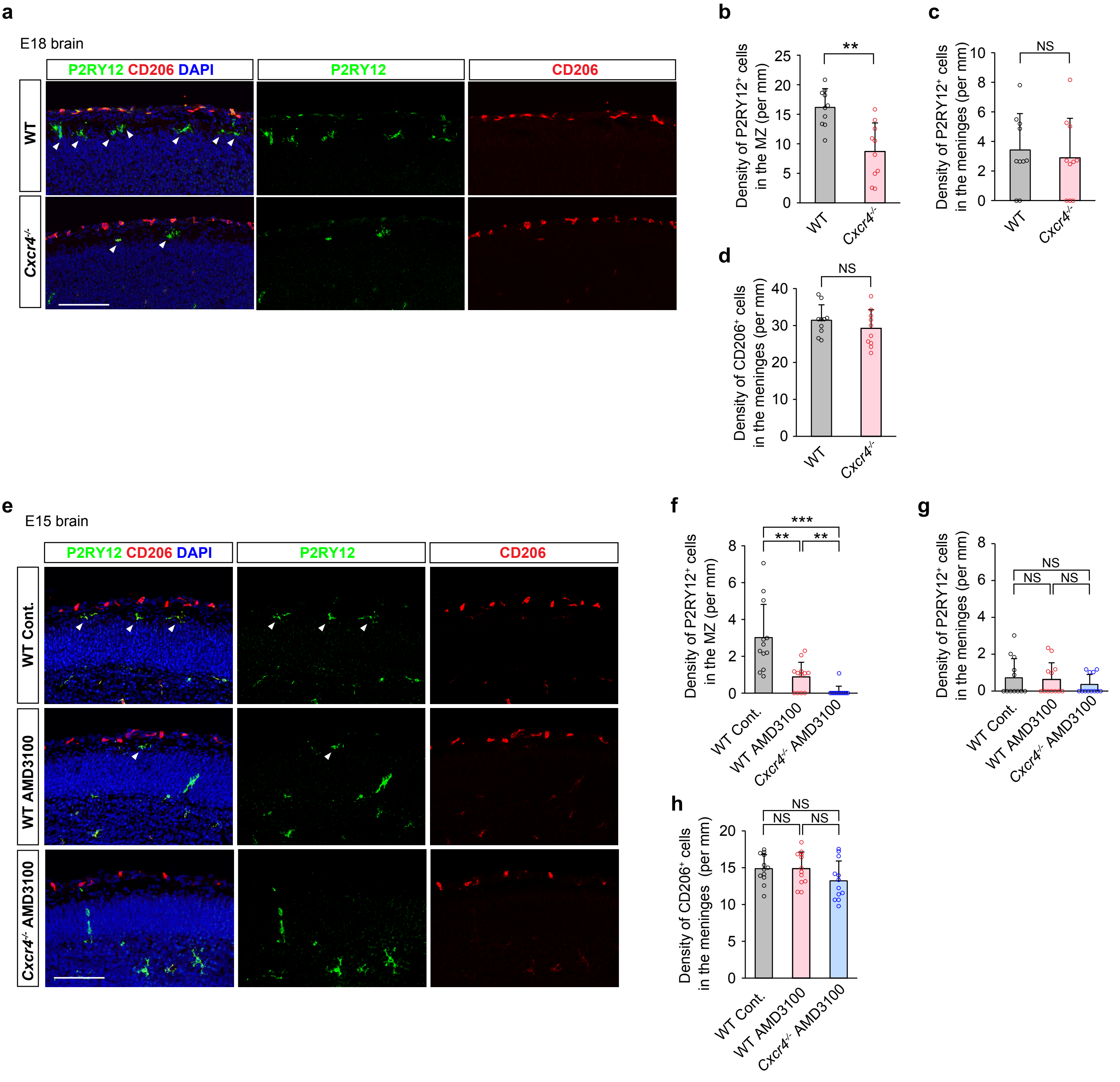

Supplementary Figure 5 The density of microglia and perivascular macrophages in the MZ and meninges
(**a**) Immunostaining for P2RY12 (green), CD206 (red) and DAPI (blue) in E18 WT and *Cxcr4*$^{-/-}$ mouse cerebral walls. Scale bar, 100 µm. (**b**–**d**) The density of P2RY12$^+$ microglia in the MZ (**b**; P = 0.0011) and the meninges (**c**; P = 0.628) and the density of CD206$^+$ macrophages in the meninges (**d**; P = 0.271) (two-sided Mann-Whitney U test; n = 10 sections from 5 mice). (**e**) Immunostaining for P2RY12 (green), CD206 (red) and DAPI (blue) in E15 cerebral walls of mock-treated WT mice (Cont.) and AMD3100-treated WT and *Cxcr4*$^{-/-}$ mice. Scale bar, 100 µm. (**f**–**h**) The density of P2RY12$^+$ microglia in the MZ (**f**; P = 0.0042 [WT Cont. vs WT AMD3100], P = 0.0079 [WT AMD3100 vs *Cxcr4*$^{-/-}$ AMD3100], $P = 4.8 \times 10^{-5}$ [WT Cont. vs *Cxcr4*$^{-/-}$ AMD3100]) and the meninges (**g**; P = 0.999 [WT Cont. vs WT AMD3100], P = 0.785 [WT AMD3100 vs *Cxcr4*$^{-/-}$ AMD3100], P = 0.785 [WT Cont. vs *Cxcr4*$^{-/-}$ AMD3100]), and the density of CD206$^+$ macrophages in the meninges (**h**; P = 0.993 [WT Cont. vs WT AMD3100], P = 0.264 [WT AMD3100 vs *Cxcr4*$^{-/-}$ AMD3100], P = 0.264 [WT Cont. vs *Cxcr4*$^{-/-}$ AMD3100]) (two-sided Steel-Dwass test; n = 12 sections from 4 mice). Data are presented as the mean values ± S.D. Source data are provided as a Source Data file.

Supplementary Figure 6

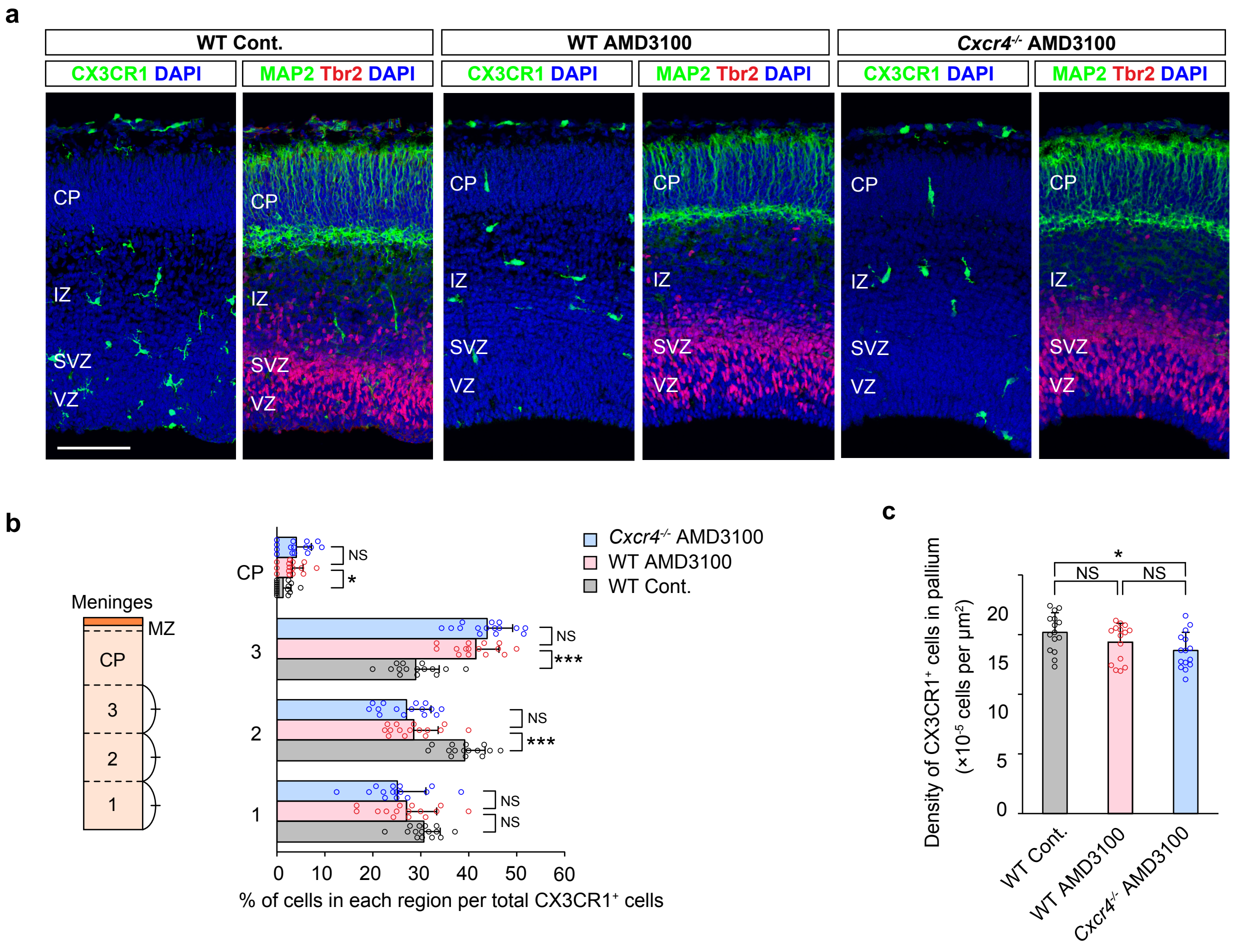

Supplementary Figure 6 The CXCL12/CXCR4 system plays a pivotal role in the microglial positioning in the cerebral wall

(**a**) Immunostaining for CX3CR1/DAPI (left) and MAP2/Tbr2/DAPI (right) in the E15 cerebral walls of mock-treated WT mice (Cont.), and AMD3100-treated WT and *Cxcr4*$^{-/-}$ mice. Scale bar, 100 μm. (**b**) Graph depicting the percentage of CX3CR1^{+} cells in each bin (two-sided Steel-Dwass test; n = 15 sections from 5 mice; For bin 1, P = 0.136 [WT Cont. vs WT AMD3100] and 0.566 [WT AMD3100 vs *Cxcr4*$^{-/-}$ AMD3100]. For bin 2, P = 1.2 × 10^{-4} [WT Cont. vs WT AMD3100] and 0.748 [WT AMD3100 vs *Cxcr4*$^{-/-}$ AMD3100]. For bin 3, P = 3.9 × 10^{-5} [WT Cont. vs WT AMD3100] and 0.426 [WT AMD3100 vs *Cxcr4*$^{-/-}$ AMD3100]. For bin CP, P = 0.024 [WT Cont. vs WT AMD3100] and 0.459 [WT AMD3100 vs *Cxcr4*$^{-/-}$ AMD3100]). (**c**) Graph showing the density of pallial CX3CR1^{+} cells (two-sided Steel-Dwass test; n = 15 sections from 5 mice; P = 0.346 [WT Cont. vs WT AMD3100], 0.515 [WT AMD3100 vs *Cxcr4*$^{-/-}$ AMD3100] and P = 0.045 [WT Cont. vs *Cxcr4*$^{-/-}$ AMD3100]). Data are presented as the mean values ± S.D. Source data are provided as a Source Data file.

Supplementary Figure 7

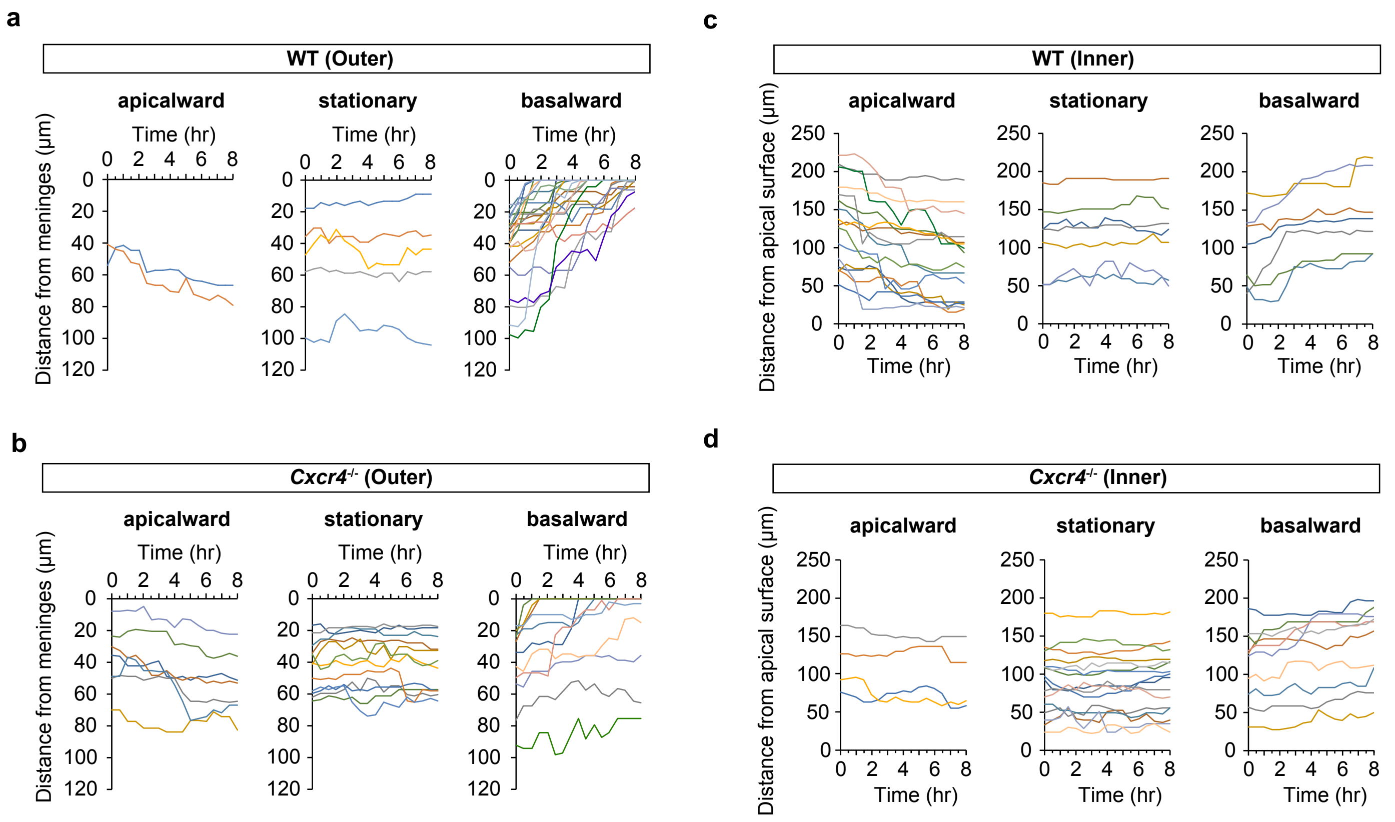

Supplementary Figure 7 The trajectories of microglia in *Cxcr4*$^{-/-}$ mouse cortical slices
(**a**–**d**) The trajectories of microglia in the outer (**a**, **b**) and inner (**c**, **d**) cerebral walls of WT (**a**, **c**) and *Cxcr4*$^{-/-}$ mice (**b**, **d**). Source data are provided as a Source Data file.

Supplementary Figure 8

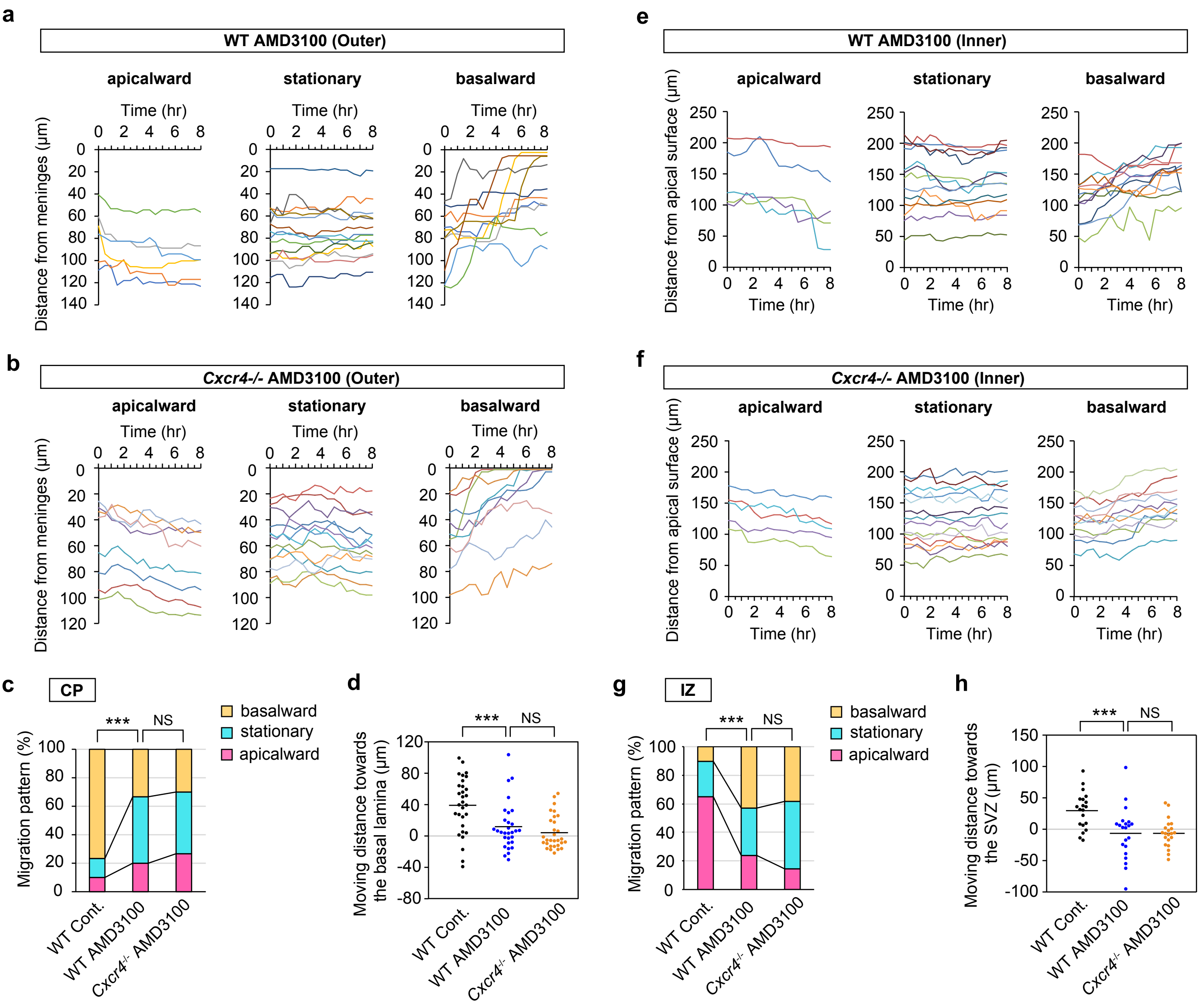

Supplementary Figure 8 The trajectories and the translocation patterns of microglia in AMD3100-treated cortical slices
(**a**, **b**, **e**, **f**) The trajectories of microglia initially positioned in the outer (**a**, **b**) and inner (**e**, **f**) region of WT mouse slices (**a**, **e**) and *Cxcr4*$^{-/-}$ mouse slices (**b**, **f**), both of which were cultured in the presence of AMD3100 at 30-min intervals for 8 hr. The data are divided into three graphs according to the microglial migratory direction: apicalward, stationary, and basalward. (**c**) A comparison of translocation patterns of the microglia in the CP of mock (Cont.) and AMD3100-treated WT mouse slices and AMD3100-treated *Cxcr4*$^{-/-}$ mouse slices (two-sided Pearson' s chi-squared test; n = 30 cells in each group; $P = 3.5 \times 10^{-9}$ [WT Cont. vs WT AMD3100], P = 0.535 [WT AMD3100 vs *Cxcr4*$^{-/-}$ AMD3100], $P = 3.0 \times 10^{-10}$ [WT Cont. vs *Cxcr4*$^{-/-}$ AMD3100]). (**d**) The migratory distance of microglia in the CP towards the meninges (two-sided Steel-Dwass test; n = 30 cells in each group; P = 0.0057 [WT Cont. vs WT AMD3100], P = 0.527 [WT AMD3100 vs *Cxcr4*$^{-/-}$ AMD3100], $P = 2.1 \times 10^{-4}$ [WT Cont. vs *Cxcr4*$^{-/-}$ AMD3100]). (**g**) The translocation patterns of microglia in the IZ (two-sided Pearson' s chi-squared test; n = 20 [WT Cont.], 21 [WT AMD3100], 21 [*Cxcr4*$^{-/-}$ AMD3100] cells; $P = 1.4 \times 10^{-9}$ [WT Cont. vs WT AMD3100], P = 0.075 [WT AMD3100 vs *Cxcr4*$^{-/-}$ AMD3100], $P = 7.3 \times 10^{-13}$ [WT Cont. vs *Cxcr4*$^{-/-}$ AMD3100]). (**h**) The migratory distance of microglia in the IZ towards the apical surface (two-sided Steel-Dwass test; n = 20 [WT Cont.)], 21 [WT AMD3100], 21 [*Cxcr4*$^{-/-}$ AMD3100] cells; P = 0.0076 [WT Cont. vs WT AMD3100], P = 0.938 [WT AMD3100 vs *Cxcr4*$^{-/-}$ AMD3100], $P = 9.3 \times 10^{-4}$ [WT Cont. vs *Cxcr4*$^{-/-}$ AMD3100]). Source data are provided as a Source Data file.

Supplementary Figure 9

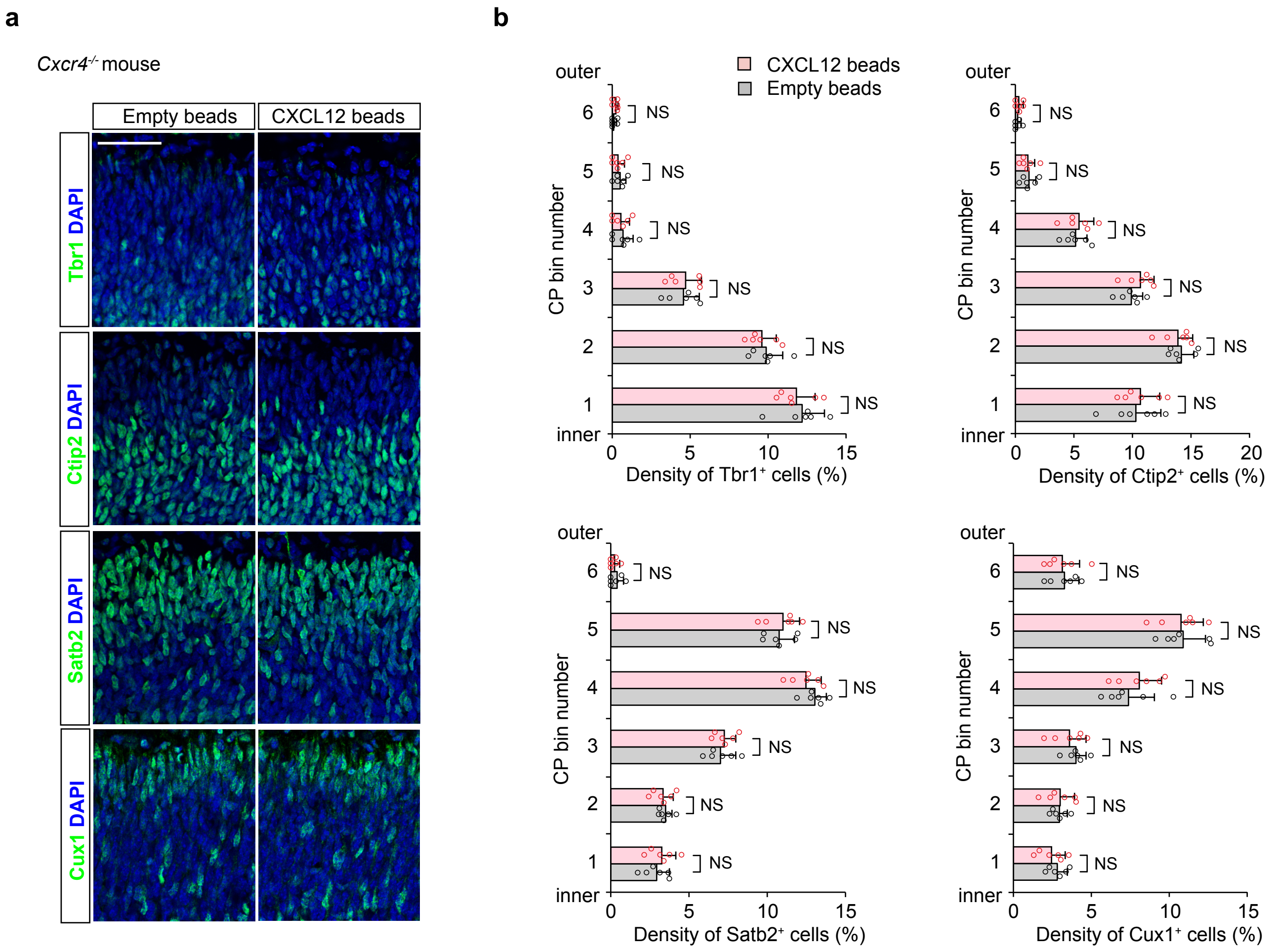

Supplementary Figure 9 Immunostaining of *Cxcr4*$^{-/-}$ mouse cortical slices on which CXCL12 beads were placed
(**a**) Representative immunostaining for Tbr1/Ctip2/Satb2/Cux1 (green) and DAPI (blue) in cortical slices derived from E15 *Cxcr4*$^{-/-}$ mice 24 hr after starting culture. Empty- or CXCL12-beads were placed on the basal part of the cortical slices. Scale bar, 50 µm. (**b**) The density of cells expressing each marker per total (DAPI$^+$) cells in 20-µm bins in the CP, which was divided into six zones and numbered from the inside, compared to that in cultured cortical slices on which empty- or CXCL12-beads were placed. The density of cells expressing each marker was comparable between slices treated with empty and CXCL12 beads, indicating that CXCL12 may not influence postmigratory cortical neurons in the expression of these transcription factors (two-sided Mann-Whitney U test; n = 6 sections from 3 mice; For Tbr1, P = 0.589, 0.699, 0.818, 0.922, 0.699 and 0.372 [from bin 1 to 6]. For Ctip2, P = 0.818, 0.818, 0.240, 0.937, 0.937 and 0.470. For Satb2, P = 0.485, 0.853, 0.623, 0.240, 0.818 and 0.870. For Cux1, P = 0.485, 0.937, 0.699, 0.485, 0.937 and 0.699). Data are presented as the mean values ± S.D. Source data are provided as a Source Data file.

Supplementary Figure 10

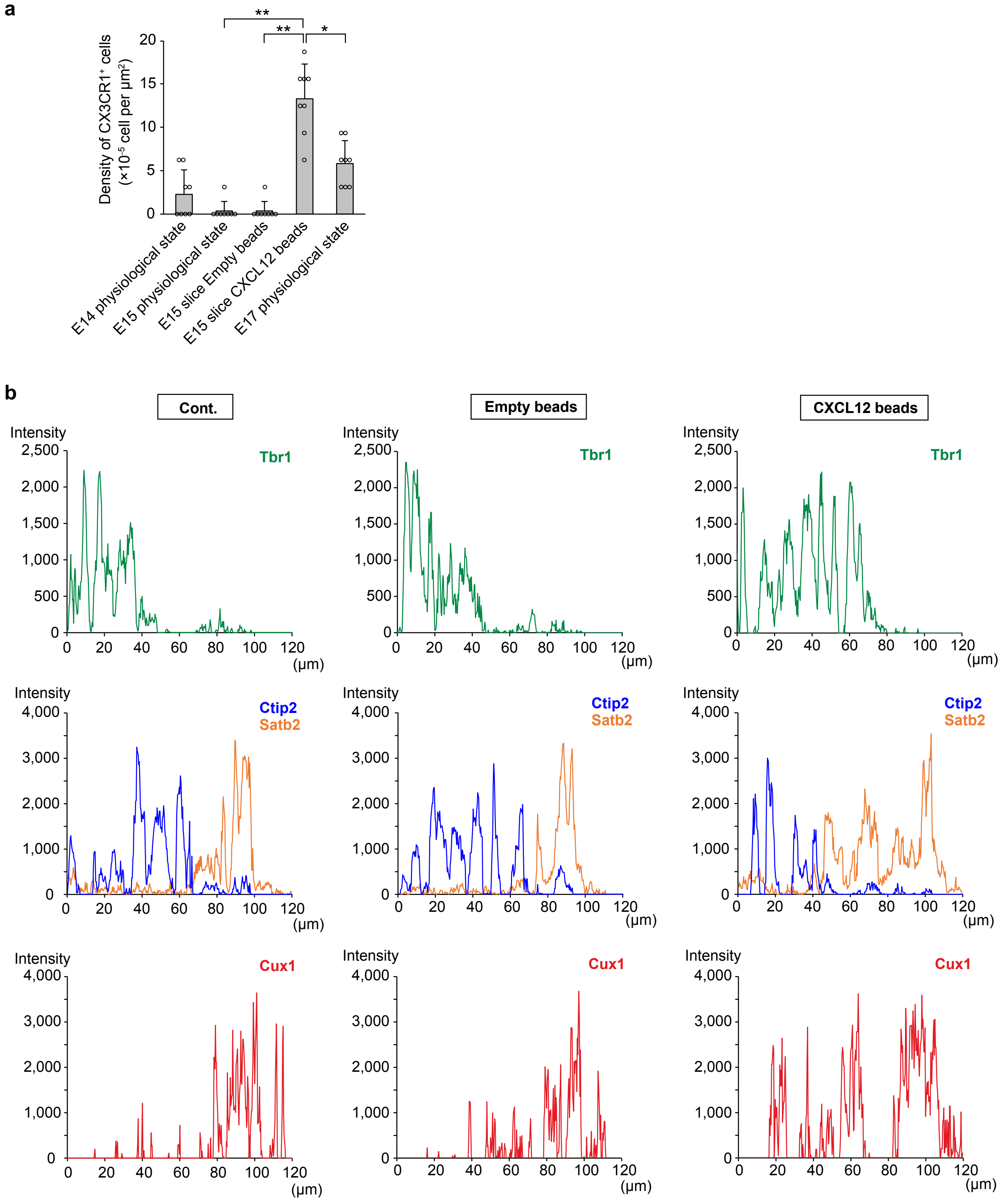

Supplementary Figure 10 Artificial delivery of microglia into the CP of cultured slices using CXCL12-soaked beads
(**a**) Comparing the density of CX3CR1⁺ cells in the CP region between E15 cultured slices on which beads were placed and brains in physiological condition at E14, E15 and E17. The density was determined by counting the number of cells inside an area of about 32,000 µm² (two-sided Steel-Dwass test; n = 8 sections; $P = 3.9 \times 10^{-3}$ [E15 physiological state vs E15 slice CXCL12 beads], $P = 3.9 \times 10^{-3}$ [E15 slice Empty beads vs E15 slice CXCL12 beads] and $P = 0.027$ [E15 slice CXCL12 beads vs E17 physiological state]). Data are presented as the mean values ± S.D. (**b**) Line scans for the fluorescent intensity of Tbr1, Ctip2, Satb2 and Cux1 expression in cultured slices on which empty or CXCL12 beads were placed and in control slices without beads (**Fig. 4f**). Source data are provided as a Source Data file.

Supplementary Figure 11

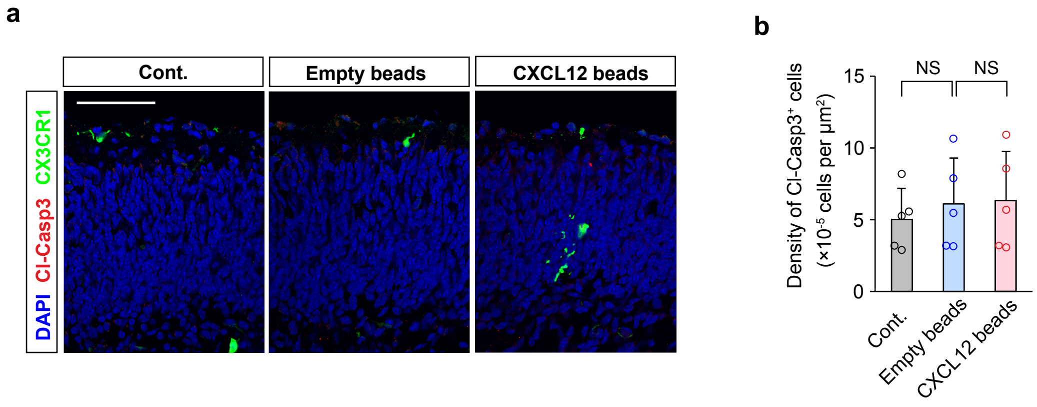

Supplementary Figure 11 Immunostaining of Cl-Casp-3 in cultured slices for evaluation of cell viability
(**a**) Immunostaining for CX3CR1 (green), Cl-Casp-3 (red) and DAPI (blue) in cultured slices on which empty or CXCL12 beads were placed and in control slices without beads. Scale bar, 100 µm. (**b**) A comparison of the number of Cl-Casp-3$^+$ cells per total (DAPI$^+$) cells. We did not detect a significant difference between that in the control and empty beads-treated slices, and between that in the empty beads-treated and CXCL12 beads-treated slices (two-sided Steel-Dwass test; *n* = 5 sections from independent slices; *P* = 0.947 [Cont. vs Empty beads], *P* = 0.947 [empty beads vs CXCL12 beads], *P* = 0.615 [Cont. vs CXCL12 beads]), indicating that the cell viability in the slices was comparable among all samples. Data are presented as the mean values ± S.D. Source data are provided as a Source Data file.

Supplementary Figure 12

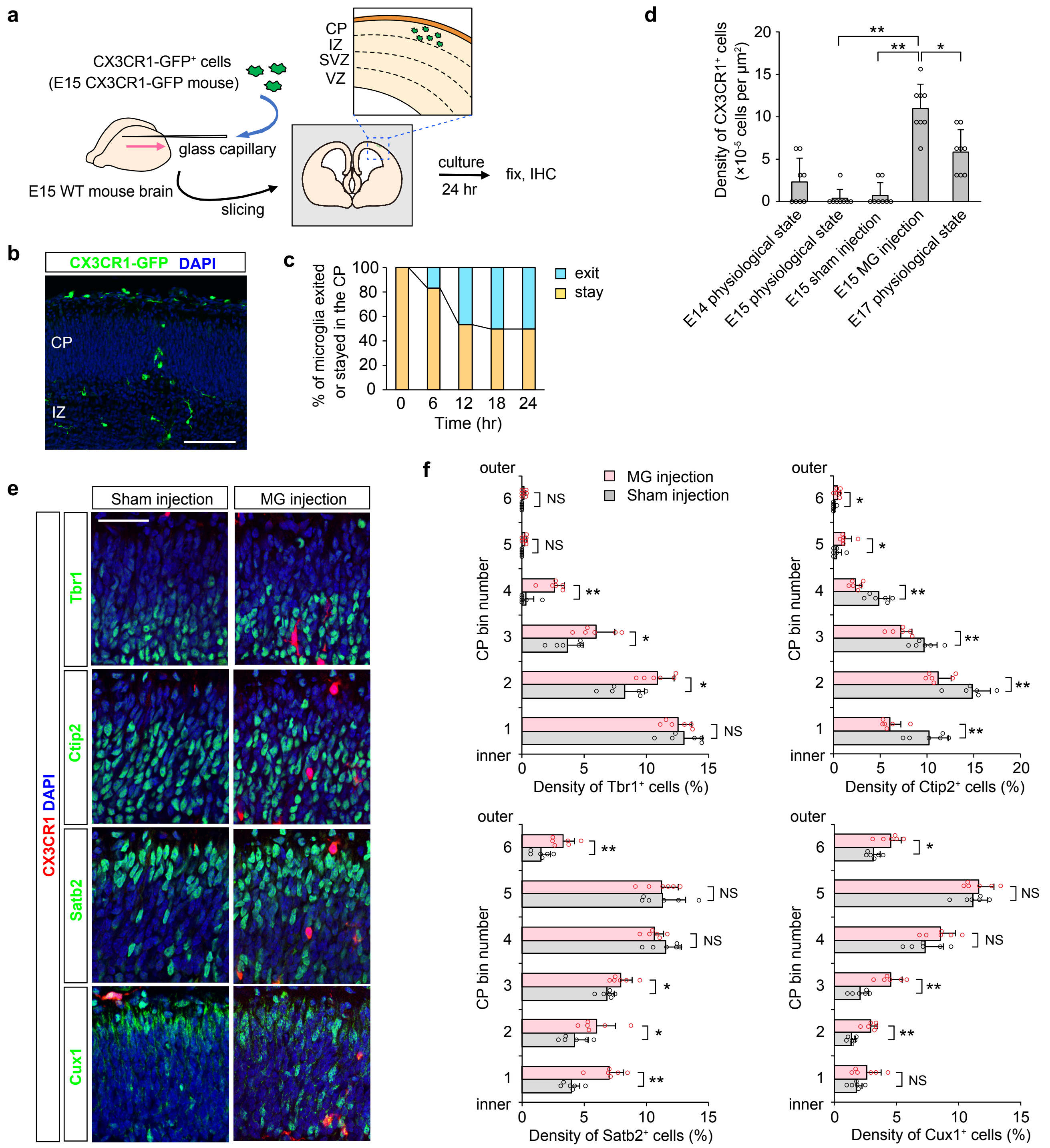

Supplementary Figure 12 Microglial transplantation into the CP of region the cortical slices

(**a**) The experimental design of the transplantation of isolated E15 CX3CR1-GFP⁺ microglia into the CP of E15 WT mouse brains. (**b**) Immunostaining for anti-GFP (CX3CR1) and DAPI in E15 mouse brains, which were fixed as soon as CX3CR1-GFP⁺ microglia were transplanted. Scale bar, 100 µm. (**c**) The ratio of microglia that stayed in or exited the CP was monitored for 24 hr. “Exit” and “stay” meant microglia exited and stayed in the CP, respectively. (**d**) Comparing the density of CX3CR1⁺ cells in the CP region between E15 cultured slices into which CX3CR1⁺ cells were transplanted and brains in physiological condition at E14, E15 and E17. The density was determined by counting the number of cells inside an area of about 32,000 µm² (two-sided Steel-Dwass test; n = 8 sections; $P = 3.8 \times 10^{-3}$ [E15 physiological state vs E15 MG injection], $P = 4.6 \times 10^{-3}$ [E15 sham injection vs E15 MG injection] and P = 0.046 [E15 microglial injection vs E17 physiological state]). (**e**) Representative triple immunostaining (shown in pseudo color) for CX3CR1 (red), Tbr1/Ctip2/Satb2/Cux1 (green) and DAPI (blue) in brains that were injected with microglial suspension (MG injection) or only solvent (sham injection). Scale bar, 50 µm. (**f**) The density of cells expressing each marker per total (DAPI⁺) cells in 20-µm bins in the CP, which was divided into six zones and numbered from the inside, compared to that in the MG and sham injection groups (two-sided Mann-Whitney U test; n = 6 sections from 3 mice; For Tbr1, P = 0.394, 0.026, 0.015, 4.3×10^{-3}, 0.061 and 0.182 [from bin 1 to 6]. For Ctip2, P = 8.7 × 10-3, 8.7×10^{-3}, 8.7×10^{-3}, 2.2×10^{-3}, 0.041 and 0.041. For Satb2, $P = 4.3 \times 10^{-3}$, 4.1×10^{-3}, 0.026, 0.180, 0.937 and 8.7×10^{-3}. For Cux1, P = 0.301, 2.2×10^{-3}, 2.2×10^{-3}, 0.145, 0.818 and 0.015). Data are presented as the mean values ± S.D. Source data are provided as a Source Data file.

Supplementary Figure 13

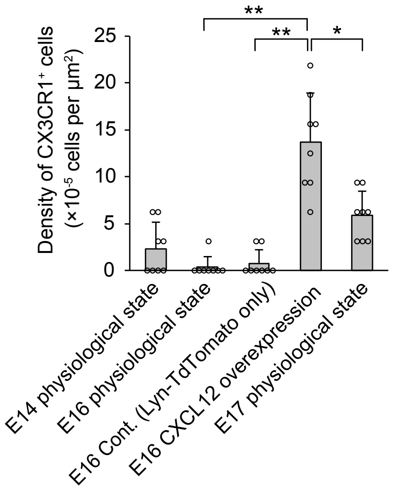

Supplementary Figure 13 The density of microglia in the CP region in which CXCL12 were overexpressed
Comparing the density of CX3CR1⁺ cells in the CP region between E16 IUE-performed brains and those in physiological condition at E14, E16 and E17. The density was determined by counting the number of cells inside an area of about 32,000 μm^2 (two-sided Steel-Dwass test; n = 8 sections; $P = 4.0 \times 10^{-3}$ [E16 physiological state vs E16 Cont. Lyn-TdTomato only], $P = 4.9 \times 10^{-3}$ [E16 Cont. Lyn-TdTomato only vs E16 CXCL12 overexpression] and P = 0.037 [E16 CXCL12 overexpression vs E17 physiological state]). Data are presented as the mean values ± S.D. Source data are provided as a Source Data file.

Supplementary Figure 14

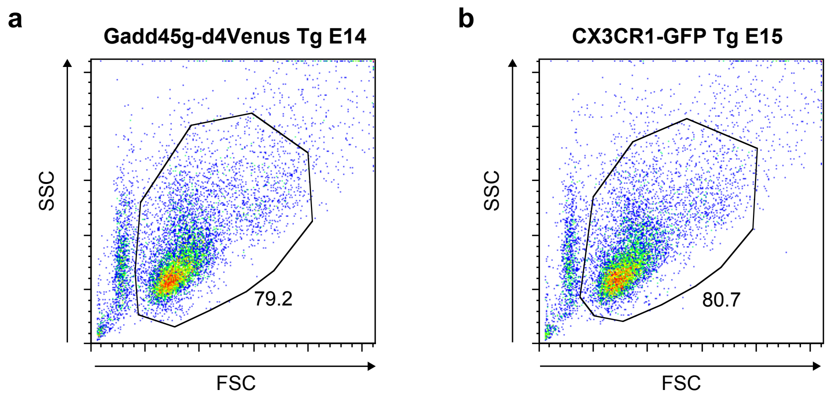

Supplementary Figure 14 The gating strategies on FACS for Gadd45g-d4Venus⁺ or CX3CR1-GFP⁺ cell isolation
The FSC/SSC plots of cerebral wall cells collected from Gadd45g-d4Venus Tg E14 mice (left) and CX3CR1-GFP E15 mice (right). The cell population was gated (black circle) on the FSC/SSC plot to remove debris and dead cells.

Supplementary Figure 15

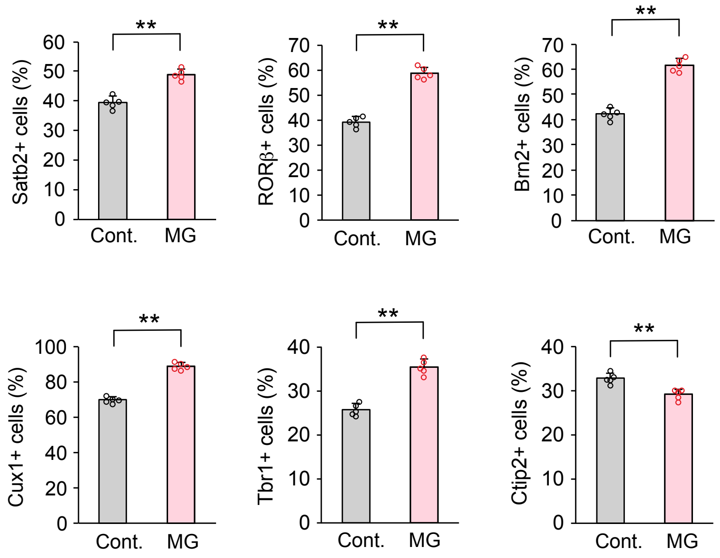

Supplementary Figure 15 Single-staining FACS analysis for the frequency of cells positive for each transcription factor Single-staining analysis for neuronal layer markers between neurons^{Cont} and neurons^{MG} groups. Graphs depicting the average percentage of Satb2$^+$, RORβ$^+$, Brn2$^+$, Cux1$^+$, Tbr1$^+$ and Ctip2$^+$ cells (two-sided Mann-Whitney U test; n = 5 independent cultures; $P = 7.9 \times 10^{-3}$ for Satb2$^+$ cells, $P = 7.9 \times 10^{-3}$ for RORβ$^+$ cells, $P = 7.9 \times 10^{-3}$ for Brn2$^+$ cells, $P = 7.9 \times 10^{-3}$ for Cux1$^+$ cells, $P = 7.9 \times 10^{-3}$ for Tbr1$^+$ cells and $P = 7.9 \times 10^{-3}$ for Ctip2$^+$ cells). Data are presented as the mean values ± S.D. Source data are provided as a Source Data file.

Supplementary Figure 16

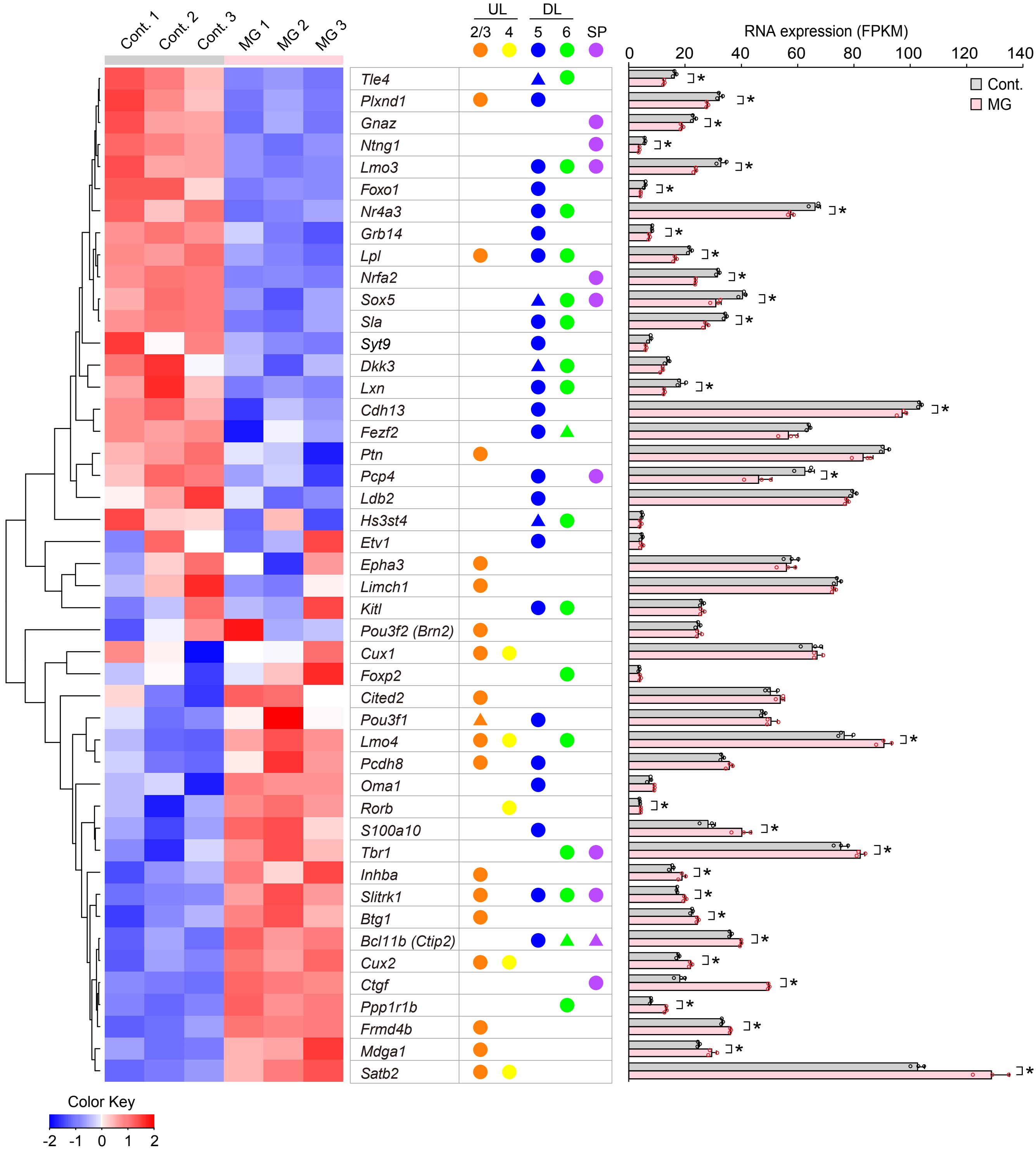

Supplementary Figure 16 A comparison of the FPKM values of 46 principal genes in RNA-Seq analysis
A comparison of the FPKM values of 46 representative genes, which are uniquely expressed in particular cortical layer(s) and important for neuronal differentiation, between the neurons^{Cont} and neurons^{MG} groups (*n* = 3 samples obtained from independent cell cultures). FDR < 0.1 is considered significant (*). The middle panel shows the cortical layer identity (L2/3, L4, L5, L6 and SP [subplate]) of each gene. Circle shows that the gene is known to be strongly expressed in the corresponding layer; triangle shows its weak expression. Data are presented as the mean values ± S.D. Source data and the exact FDR values are provided as a Source Data file and Supplementary Data 1.

Supplementary Figure 17

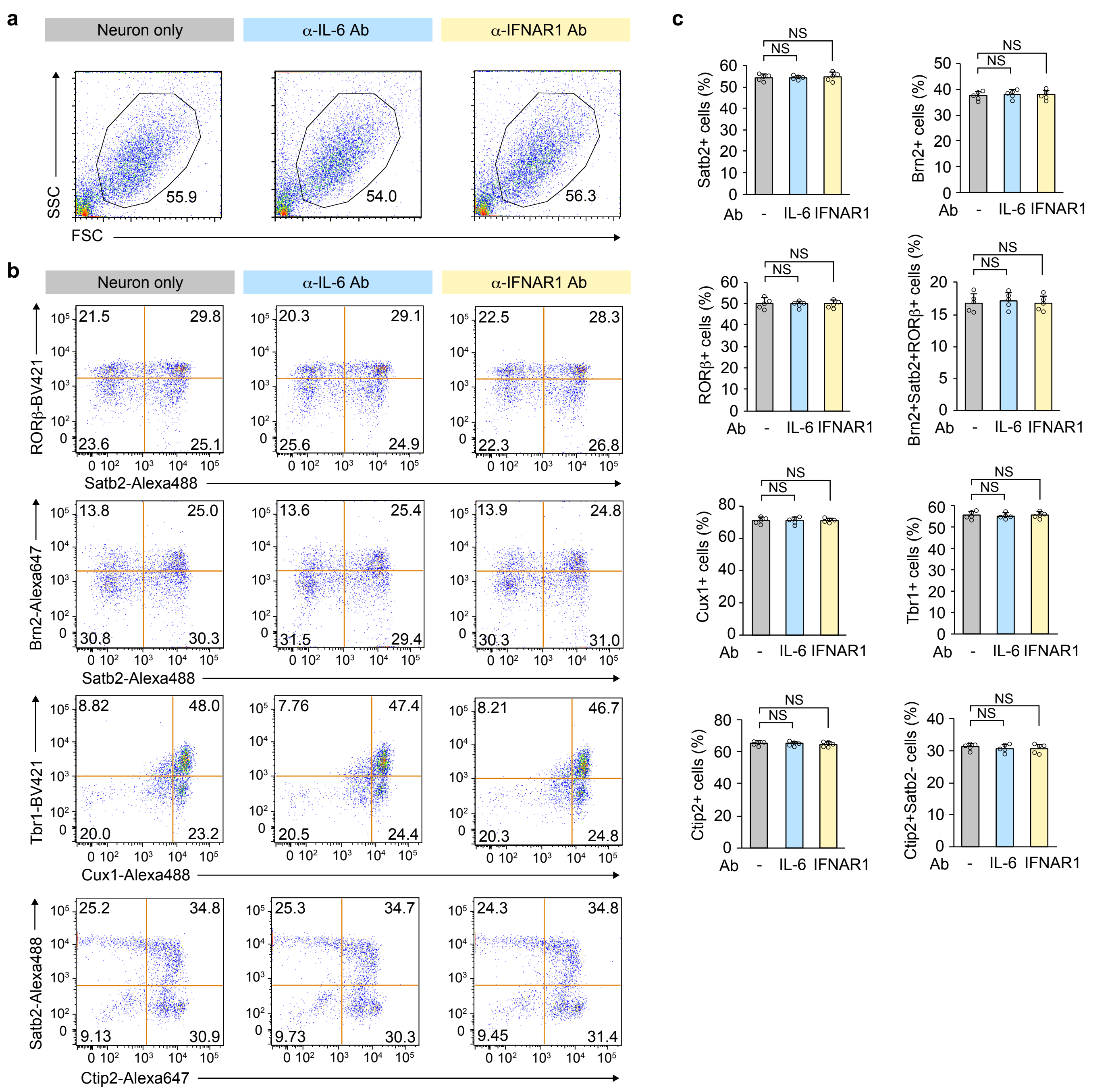

Supplementary Figure 17 Neutralizing antibodies themselves did not affect the expression of neuronal subtype-associated transcription factors

(**a**) The plots show a FSC/SSC gating strategy of in vitro-prepared neurons cultured with/without neutralizing antibodies. (**b**) Representative FACS analysis data of in vitro-prepared CP-like neurons 24 hr after culturing in the presence of neutralizing antibodies for IL-6 or IFNAR1 without microglia showing the proportion of Brn2$^+$, RORβ$^+$, Satb2$^+$, Cux1$^+$, Tbr1$^+$ and Ctip2$^+$ cells. (**c**) Graphs depicting the average percentage of Satb2$^+$, Brn2$^+$, RORβ$^+$, Brn2$^+$Satb2$^+$RORβ$^+$, Cux1$^+$, Tbr1$^+$, Ctip2$^+$ and Ctip2$^+$Satb2$^-$ cells (two-sided Steel-Dwass test; n = 5 independent cultures; P = 0.947 and 0.976 for Satb2$^+$ cells, P = 0.860 and 0.994 for Brn2$^+$ cells, P = 0.994 and 0.976 for RORβ$^+$ cells, P = 0.947 and 0.994 for Brn2$^+$Satb2$^+$RORβ$^+$ cells, P = 0.994 and 0.994 for Cux1$^+$ cells, P = 0.976 and 0.994 for Tbr1$^+$ cells, P = 0.860 and 0.745 for Ctip2$^+$ cells and P = 0.804 and 0.742 for Ctip2$^+$Satb2$^-$ cells [left to right]). Data are presented as the mean values ± S.D. Source data are provided as a Source Data file.

Supplementary Figure 18

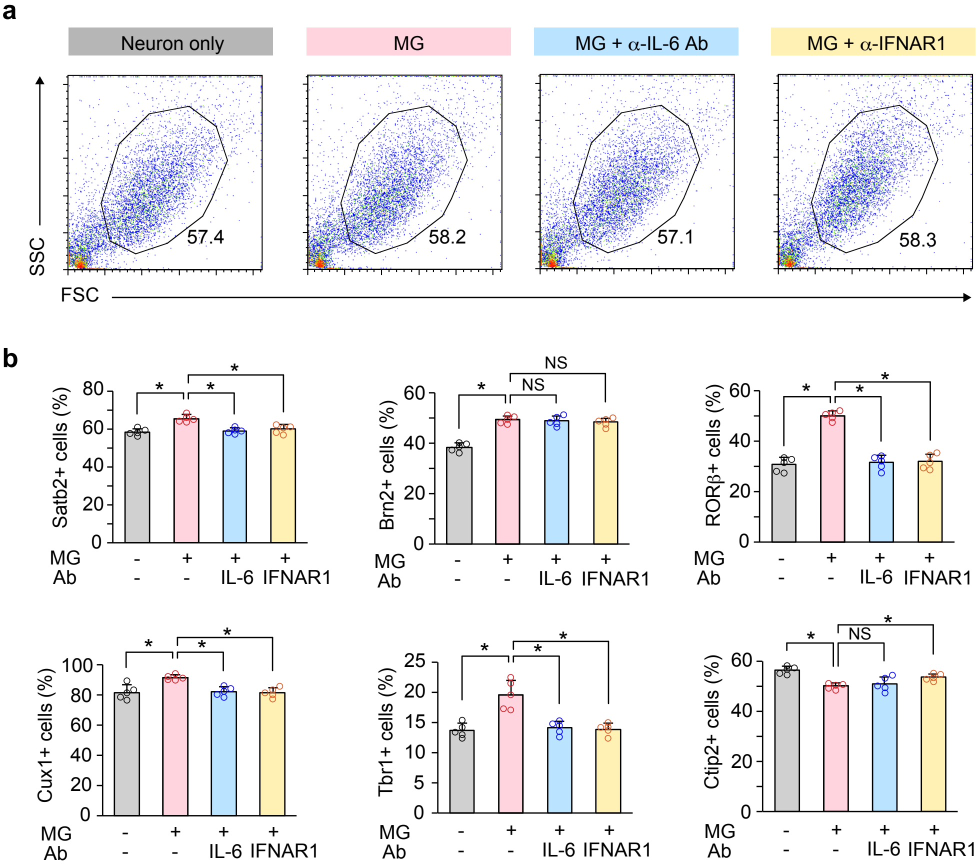

Supplementary Figure 18 Single-staining FACS analysis for the frequency of cells positive for each transcription factor in neutralizing antibody-treated CP-like neurons

(**a**) The plots show a FSC/SSC gating strategy of in vitro-prepared neurons cultured with/without microglia and neutralizing antibodies. (**b**) Single-staining analysis for neuronal layer markers in neurons that had been cocultured with microglia in the presence of neutralizing antibodies for IL-6 or IFNAR1. Graphs depicting the average percentage of Satb2⁺, Brn2⁺, RORβ⁺, Cux1⁺, Tbr1⁺ and Ctip2⁺ cells (two-sided Steel-Dwass test; n = 5 independent cultures; P = 0.045, 0.045 and 0.045 for Satb2⁺ cells, P = 0.045, 0.989 and 0.783 for Brn2⁺ cells, P = 0.045, 0.045 and 0.045 for RORβ⁺ cells, P = 0.045, 0.045 and 0.045 for Cux1⁺ cells, P = 0.045, 0.045 and 0.045 for Tbr1⁺ cells, and P = 0.045, 0.989 and 0.045 for Ctip2⁺ cells [in the order of control vs microglia-added samples without antibodies, microglia-added samples without antibodies vs those treated with anti-IL-6 antibodies, and microglia-added samples without antibodies vs those treated with anti-IFNAR1 antibodies]). Data are presented as the mean values ± S.D. Source data are provided as a Source Data file.

Supplementary Figure 19

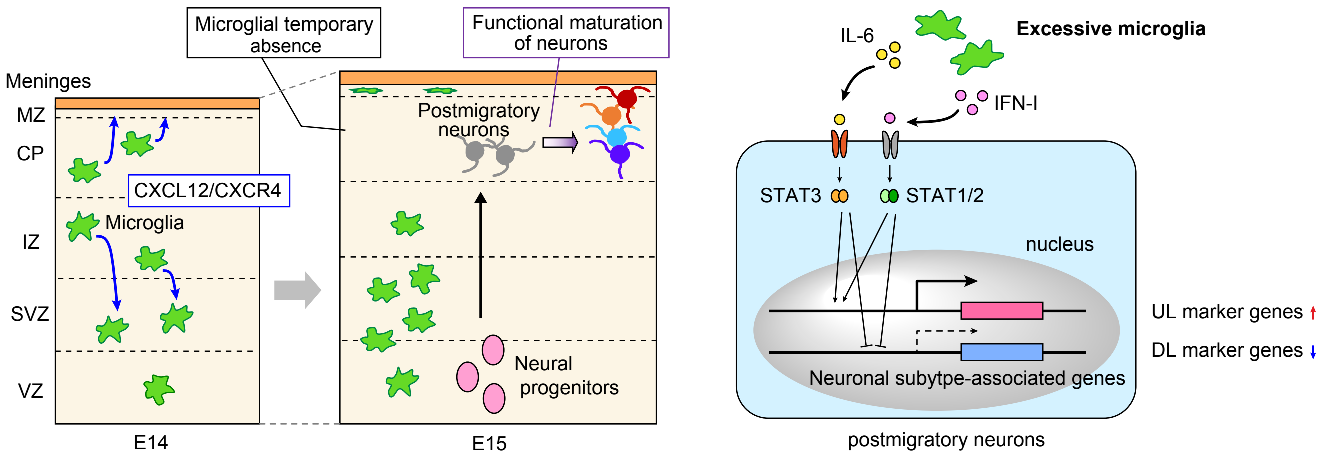

Supplementary Figure 19 The model proposed in this study

We demonstrated that microglia transiently exit the midembryonic CP via the CXCL12/CXCR4-mediated bidirectional attraction system. If microglia inadvertently colonize the CP, they would destabilize the expression properties of subtype-associated transcription factors in postmigratory neurons, inducing a reduction of the expression of DL marker genes and an increase of typical UL marker genes. Further, we found that microglia-derived IL-6 and IFN-I are two important mediators which participate in the disturbance of the expression of neuronal subtype-associated genes. Hence, the developing cortex expels microglia from the midembryonic CP to appropriately fine-tune the expression of molecules needed for proper differentiation of postmigratory neurons, thus securing the establishment of functional cortical circuit.

Supplementary Table 1

Figure	Marker	Type of test	*P*-value
Fig. 4g	Tbr1	two-sided Steel-Dwass test	bin 1: *P* = 0.994 (Cont. vs Empty beads), *P* = 0.545 (Empty beads vs CXCL12 beads)
			bin 2: *P* = 0.976 (Cont. vs Empty beads), *P* = 0.907 (Empty beads vs CXCL12 beads)
			bin 3: *P* = 0.994 (Cont. vs Empty beads), *P* = 0.024 (Empty beads vs CXCL12 beads)
			bin 4: *P* = 0.959 (Cont. vs Empty beads), *P* = 2.2 × 10^{-3} (Empty beads vs CXCL12 beads)
			bin 5: *P* = 0.995 (Cont. vs Empty beads), *P* = 1.3 × 10^{-3} (Empty beads vs CXCL12 beads)
			bin 6: *P* = 0.577 (Cont. vs Empty beads), *P* = 0.221 (Empty beads vs CXCL12 beads)
	Ctip2	two-sided Steel-Dwass test	bin 1: *P* = 0.859 (Cont. vs Empty beads), *P* = 4.6 × 10^{-3} (Empty beads vs CXCL12 beads)
			bin 2: *P* = 0.678 (Cont. vs Empty beads), *P* = 2.2 × 10^{-3} (Empty beads vs CXCL12 beads)
			bin 3: *P* = 0.859 (Cont. vs Empty beads), *P* = 4.6 × 10^{-3} (Empty beads vs CXCL12 beads)
			bin 4: *P* = 0.859 (Cont. vs Empty beads), *P* = 0.024 (Empty beads vs CXCL12 beads)
			bin 5: *P* = 0.976 (Cont. vs Empty beads), *P* = 0.305 (Empty beads vs CXCL12 beads)
			bin 6: *P* = 0.677 (Cont. vs Empty beads), *P* = 0.710 (Empty beads vs CXCL12 beads)
	Satb2	two-sided Steel-Dwass test	bin 1: *P* = 0.947 (Cont. vs Empty beads), *P* = 9.2 × 10^{-3} (Empty beads vs CXCL12 beads)
			bin 2: *P* = 0.448 (Cont. vs Empty beads), *P* = 2.2 × 10^{-3} (Empty beads vs CXCL12 beads)
			bin 3: *P* = 0.418 (Cont. vs Empty beads), *P* = 0.054 (Empty beads vs CXCL12 beads)
			bin 4: *P* = 0.743 (Cont. vs Empty beads), *P* = 0.359 (Empty beads vs CXCL12 beads)
			bin 5: *P* = 0.480 (Cont. vs Empty beads), *P* = 0.418 (Empty beads vs CXCL12 beads)
			bin 6: *P* = 0.998 (Cont. vs Empty beads), *P* = 9.2 × 10^{-3} (Empty beads vs CXCL12 beads)
	Cux1	two-sided Steel-Dwass test	bin 1: *P* = 0.832 (Cont. vs Empty beads), *P* = 0.017 (Empty beads vs CXCL12 beads)
			bin 2: *P* = 0.859 (Cont. vs Empty beads), *P* = 6.6 × 10^{-3} (Empty beads vs CXCL12 beads)
			bin 3: *P* = 0.832 (Cont. vs Empty beads), *P* = 0.013 (Empty beads vs CXCL12 beads)
			bin 4: *P* = 0.710 (Cont. vs Empty beads), *P* = 0.804 (Empty beads vs CXCL12 beads)
			bin 5: *P* = 0.928 (Cont. vs Empty beads), *P* = 0.907 (Empty beads vs CXCL12 beads)
			bin 6: *P* = 0.678 (Cont. vs Empty beads), *P* = 2.2 × 10^{-3} (Empty beads vs CXCL12 beads)
Fig. 4l	Tbr1	two-sided Steel-Dwass test	bin 1: *P* = 0.907 (Cont. vs CXCL12), *P* = 0.963 (CXCL12 vs CXCL12 with CL)
			bin 2: *P* = 0.141 (Cont. vs CXCL12), *P* = 0.928 (CXCL12 vs CXCL12 with CL)
			bin 3: *P* = 4.6 × 10^{-3} (Cont. vs CXCL12), *P* = 0.036 (CXCL12 vs CXCL12 with CL)
			bin 4: *P* = 0.015 (Cont. vs CXCL12), *P* = 0.042 (CXCL12 vs CXCL12 with CL)
			bin 5: *P* = 0.054 (Cont. vs CXCL12), *P* = 0.280 (CXCL12 vs CXCL12 with CL)
			bin 6: *P* = 2.2 × 10^{-3} (Cont. vs CXCL12), *P* = 0.020 (CXCL12 vs CXCL12 with CL)
			bin 7: *P* = 0.965 (Cont. vs CXCL12), *P* = 0.866 (CXCL12 vs CXCL12 with CL)
			bin 8: *P* = 0.154 (Cont. vs CXCL12), *P* = 0.154 (CXCL12 vs CXCL12 with CL)
	Ctip2	two-sided Steel-Dwass test	bin 1: *P* = 0.331 (Cont. vs CXCL12), *P* = 0.907 (CXCL12 vs CXCL12 with CL)
			bin 2: *P* = 3.2 × 10^{-3} (Cont. vs CXCL12), *P* = 4.6 × 10^{-3} (CXCL12 vs CXCL12 with CL)
			bin 3: *P* = 3.2 × 10^{-3} (Cont. vs CXCL12), *P* = 0.036 (CXCL12 vs CXCL12 with CL)
			bin 4: *P* = 0.986 (Cont. vs CXCL12), *P* = 0.710 (CXCL12 vs CXCL12 with CL)
			bin 5: *P* = 0.907 (Cont. vs CXCL12), *P* = 0.418 (CXCL12 vs CXCL12 with CL)
			bin 6: *P* = 0.027 (Cont. vs CXCL12), *P* = 0.479 (CXCL12 vs CXCL12 with CL)
			bin 7: *P* = 0.126 (Cont. vs CXCL12), *P* = 0.677 (CXCL12 vs CXCL12 with CL)
			bin 8: *P* = 0.882 (Cont. vs CXCL12), *P* = 0.801 (CXCL12 vs CXCL12 with CL)
	Satb2	two-sided Steel-Dwass test	bin 1: *P* = 4.6 × 10^{-3} (Cont. vs CXCL12), *P* = 0.031 (CXCL12 vs CXCL12 with CL)
			bin 2: *P* = 2.2 × 10^{-3} (Cont. vs CXCL12), *P* = 2.2 × 10^{-3} (CXCL12 vs CXCL12 with CL)
			bin 3: *P* = 0.031 (Cont. vs CXCL12), *P* = 3.2 × 10^{-3} (CXCL12 vs CXCL12 with CL)
			bin 4: *P* = 0.804 (Cont. vs CXCL12), *P* = 0.803 (CXCL12 vs CXCL12 with CL)
			bin 5: *P* = 0.832 (Cont. vs CXCL12), *P* = 0.773 (CXCL12 vs CXCL12 with CL)
			bin 6: *P* = 0.101 (Cont. vs CXCL12), *P* = 0.213 (CXCL12 vs CXCL12 with CL)
			bin 7: *P* = 3.2 × 10^{-3} (Cont. vs CXCL12), *P* = 9.2 × 10^{-3} (CXCL12 vs CXCL12 with CL)
			bin 8: *P* = 0.013 (Cont. vs CXCL12), *P* = 0.023 (CXCL12 vs CXCL12 with CL)
	Cux1	two-sided Steel-Dwass test	bin 1: *P* = 2.2 × 10^{-3} (Cont. vs CXCL12), *P* = 0.141 (CXCL12 vs CXCL12 with CL)
			bin 2: *P* = 0.054 (Cont. vs CXCL12), *P* = 0.612 (CXCL12 vs CXCL12 with CL)
			bin 3: *P* = 4.6 × 10^{-3} (Cont. vs CXCL12), *P* = 0.054 (CXCL12 vs CXCL12 with CL)
			bin 4: *P* = 0.013 (Cont. vs CXCL12), *P* = 0.678 (CXCL12 vs CXCL12 with CL)
			bin 5: *P* = 0.113 (Cont. vs CXCL12), *P* = 0.678 (CXCL12 vs CXCL12 with CL)
			bin 6: *P* = 6.6 × 10^{-3} (Cont. vs CXCL12), *P* = 0.884 (CXCL12 vs CXCL12 with CL)
			bin 7: *P* = 0.612 (Cont. vs CXCL12), *P* = 0.359 (CXCL12 vs CXCL12 with CL)
			bin 8: *P* = 0.305 (Cont. vs CXCL12), *P* = 0.804 (CXCL12 vs CXCL12 with CL)

Figure	Marker	Type of test	P-value
Fig. 10c	Tbr1	two-sided Steel-Dwass test	bin 1: $P = 0.970$ (Cont. vs CXCL12), $P = 0.978$ (CXCL12 vs CXCL12, α-IL-6 Ab), $P = 0.918$ (CXCL12 vs CXCL12, α-IFNAR1 Ab), $P = 0.993$ (CXCL12 vs CXCL12, α-IL-6/IFNAR1 Ab)
			bin 2: $P = 0.879$ (Cont. vs CXCL12), $P = 0.615$ (CXCL12 vs CXCL12, α-IL-6 Ab), $P = 0.582$ (CXCL12 vs CXCL12, α-IFNAR1 Ab), $P = 0.650$ (CXCL12 vs CXCL12, α-IL-6/IFNAR1 Ab)
			bin 3: $P = 0.020$ (Cont. vs CXCL12), $P = 0.0496$ (CXCL12 vs CXCL12, α-IL-6 Ab), $P = 0.027$ (CXCL12 vs CXCL12, α-IFNAR1 Ab), $P = 0.0496$ (CXCL12 vs CXCL12, α-IL-6/IFNAR1 Ab)
			bin 4: $P = 0.0099$ (Cont. vs CXCL12), $P = 0.043$ (CXCL12 vs CXCL12, α-IL-6 Ab), $P = 0.0496$ (CXCL12 vs CXCL12, α-IFNAR1 Ab), $P = 0.0496$ (CXCL12 vs CXCL12, α-IL-6/IFNAR1 Ab)
			bin 5: $P = 0.220$ (Cont. vs CXCL12), $P = 0.746$ (CXCL12 vs CXCL12, α-IL-6 Ab), $P = 0.268$ (CXCL12 vs CXCL12, α-IFNAR1 Ab), $P = 0.805$ (CXCL12 vs CXCL12, α-IL-6/IFNAR1 Ab)
			bin 6: $P = 0.037$ (Cont. vs CXCL12), $P = 0.197$ (CXCL12 vs CXCL12, α-IL-6 Ab), $P = 6.4 \times 10^{-3}$ (CXCL12 vs CXCL12, α-IFNAR1 Ab), $P = 0.019$ (CXCL12 vs CXCL12, α-IL-6/IFNAR1 Ab)
			bin 7: $P = 1.000$ (Cont. vs CXCL12), $P = 1.000$ (CXCL12 vs CXCL12, α-IL-6 Ab), $P = 0.999$ (CXCL12 vs CXCL12, α-IFNAR1 Ab), $P = 0.969$ (CXCL12 vs CXCL12, α-IL-6/IFNAR1 Ab)
			bin 8: $P = 0.839$ (Cont. vs CXCL12), $P = 0.346$ (CXCL12 vs CXCL12, α-IL-6 Ab), $P = 0.346$ (CXCL12 vs CXCL12, α-IFNAR1 Ab), $P = 0.346$ (CXCL12 vs CXCL12, α-IL-6/IFNAR1 Ab)
	Ctip2	two-sided Steel-Dwass test	bin 1: $P = 0.158$ (Cont. vs CXCL12), $P = 0.178$ (CXCL12 vs CXCL12, α-IL-6 Ab), $P = 0.268$ (CXCL12 vs CXCL12, α-IFNAR1 Ab), $P = 0.479$ (CXCL12 vs CXCL12, α-IL-6/IFNAR1 Ab)
			bin 2: $P = 0.020$ (Cont. vs CXCL12), $P = 0.0496$ (CXCL12 vs CXCL12, α-IL-6 Ab), $P = 0.0496$ (CXCL12 vs CXCL12, α-IFNAR1 Ab), $P = 0.037$ (CXCL12 vs CXCL12, α-IL-6/IFNAR1 Ab)
			bin 3: $P = 7.0 \times 10^{-3}$ (Cont. vs CXCL12), $P = 7.0 \times 10^{-3}$ (CXCL12 vs CXCL12, α-IL-6 Ab), $P = 0.014$ (CXCL12 vs CXCL12, α-IFNAR1 Ab), $P = 7.0 \times 10^{-3}$ (CXCL12 vs CXCL12, α-IL-6/IFNAR1 Ab)
			bin 4: $P = 0.479$ (Cont. vs CXCL12), $P = 0.220$ (CXCL12 vs CXCL12, α-IL-6 Ab), $P = 0.413$ (CXCL12 vs CXCL12, α-IFNAR1 Ab), $P = 0.582$ (CXCL12 vs CXCL12, α-IL-6/IFNAR1 Ab)
			bin 5: $P = 1.000$ (Cont. vs CXCL12), $P = 0.985$ (CXCL12 vs CXCL12, α-IL-6 Ab), $P = 0.970$ (CXCL12 vs CXCL12, α-IFNAR1 Ab), $P = 0.993$ (CXCL12 vs CXCL12, α-IL-6/IFNAR1 Ab)
			bin 6: $P = 0.049$ (Cont. vs CXCL12), $P = 0.899$ (CXCL12 vs CXCL12, α-IL-6 Ab), $P = 0.086$ (CXCL12 vs CXCL12, α-IFNAR1 Ab), $P = 0.141$ (CXCL12 vs CXCL12, α-IL-6/IFNAR1 Ab)
			bin 7: $P = 0.219$ (Cont. vs CXCL12), $P = 0.267$ (CXCL12 vs CXCL12, α-IL-6 Ab), $P = 0.442$ (CXCL12 vs CXCL12, α-IFNAR1 Ab), $P = 0.855$ (CXCL12 vs CXCL12, α-IL-6/IFNAR1 Ab)
			bin 8: $P = 1.000$ (Cont. vs CXCL12), $P = 0.873$ (CXCL12 vs CXCL12, α-IL-6 Ab), $P = 0.946$ (CXCL12 vs CXCL12, α-IFNAR1 Ab), $P = 1.000$ (CXCL12 vs CXCL12, α-IL-6/IFNAR1 Ab)
	Satb2	two-sided Steel-Dwass test	bin 1: $P = 0.0099$ (Cont. vs CXCL12), $P = 0.0496$ (CXCL12 vs CXCL12, α-IL-6 Ab), $P = 0.037$ (CXCL12 vs CXCL12, α-IFNAR1 Ab), $P = 0.037$ (CXCL12 vs CXCL12, α-IL-6/IFNAR1 Ab)
			bin 2: $P = 0.0099$ (Cont. vs CXCL12), $P = 0.0496$ (CXCL12 vs CXCL12, α-IL-6 Ab), $P = 0.027$ (CXCL12 vs CXCL12, α-IFNAR1 Ab), $P = 0.027$ (CXCL12 vs CXCL12, α-IL-6/IFNAR1 Ab)
			bin 3: $P = 0.0099$ (Cont. vs CXCL12), $P = 0.027$ (CXCL12 vs CXCL12, α-IL-6 Ab), $P = 0.014$ (CXCL12 vs CXCL12, α-IFNAR1 Ab), $P = 0.014$ (CXCL12 vs CXCL12, α-IL-6/IFNAR1 Ab)
			bin 4: $P = 0.879$ (Cont. vs CXCL12), $P = 0.141$ (CXCL12 vs CXCL12, α-IL-6 Ab), $P = 0.900$ (CXCL12 vs CXCL12, α-IFNAR1 Ab), $P = 0.832$ (CXCL12 vs CXCL12, α-IL-6/IFNAR1 Ab)
			bin 5: $P = 0.716$ (Cont. vs CXCL12), $P = 0.998$ (CXCL12 vs CXCL12, α-IL-6 Ab), $P = 0.615$ (CXCL12 vs CXCL12, α-IFNAR1 Ab), $P = 0.832$ (CXCL12 vs CXCL12, α-IL-6/IFNAR1 Ab)
			bin 6: $P = 0.141$ (Cont. vs CXCL12), $P = 0.832$ (CXCL12 vs CXCL12, α-IL-6 Ab), $P = 0.178$ (CXCL12 vs CXCL12, α-IFNAR1 Ab), $P = 0.111$ (CXCL12 vs CXCL12, α-IL-6/IFNAR1 Ab)
			bin 7: $P = 7.0 \times 10^{-3}$ (Cont. vs CXCL12), $P = 7.0 \times 10^{-3}$ (CXCL12 vs CXCL12, α-IL-6 Ab), $P = 0.043$ (CXCL12 vs CXCL12, α-IFNAR1 Ab), $P = 0.014$ (CXCL12 vs CXCL12, α-IL-6/IFNAR1 Ab)
			bin 8: $P = 0.027$ (Cont. vs CXCL12), $P = 0.0496$ (CXCL12 vs CXCL12, α-IL-6 Ab), $P = 0.020$ (CXCL12 vs CXCL12, α-IFNAR1 Ab), $P = 0.020$ (CXCL12 vs CXCL12, α-IL-6/IFNAR1 Ab)
	Cux1	two-sided Steel-Dwass test	bin 1: $P = 0.014$ (Cont. vs CXCL12), $P = 0.0496$ (CXCL12 vs CXCL12, α-IL-6 Ab), $P = 0.0496$ (CXCL12 vs CXCL12, α-IFNAR1 Ab), $P = 0.0496$ (CXCL12 vs CXCL12, α-IL-6/IFNAR1 Ab)
			bin 2: $P = 0.014$ (Cont. vs CXCL12), $P = 0.037$ (CXCL12 vs CXCL12, α-IL-6 Ab), $P = 0.027$ (CXCL12 vs CXCL12, α-IFNAR1 Ab), $P = 0.023$ (CXCL12 vs CXCL12, α-IL-6/IFNAR1 Ab)
			bin 3: $P = 7.0 \times 10^{-3}$ (Cont. vs CXCL12), $P = 0.0099$ (CXCL12 vs CXCL12, α-IL-6 Ab), $P = 0.0099$ (CXCL12 vs CXCL12, α-IFNAR1 Ab), $P = 7.0 \times 10^{-3}$ (CXCL12 vs CXCL12, α-IL-6/IFNAR1 Ab)
			bin 4: $P = 0.014$ (Cont. vs CXCL12), $P = 0.220$ (CXCL12 vs CXCL12, α-IL-6 Ab), $P = 0.027$ (CXCL12 vs CXCL12, α-IFNAR1 Ab), $P = 0.027$ (CXCL12 vs CXCL12, α-IL-6/IFNAR1 Ab)
			bin 5: $P = 0.716$ (Cont. vs CXCL12), $P = 0.716$ (CXCL12 vs CXCL12, α-IL-6 Ab), $P = 0.479$ (CXCL12 vs CXCL12, α-IFNAR1 Ab), $P = 0.446$ (CXCL12 vs CXCL12, α-IL-6/IFNAR1 Ab)
			bin 6: $P = 0.075$ (Cont. vs CXCL12), $P = 0.615$ (CXCL12 vs CXCL12, α-IL-6 Ab), $P = 0.322$ (CXCL12 vs CXCL12, α-IFNAR1 Ab), $P = 0.141$ (CXCL12 vs CXCL12, α-IL-6/IFNAR1 Ab)
			bin 7: $P = 0.513$ (Cont. vs CXCL12), $P = 0.777$ (CXCL12 vs CXCL12, α-IL-6 Ab), $P = 0.322$ (CXCL12 vs CXCL12, α-IFNAR1 Ab), $P = 0.582$ (CXCL12 vs CXCL12, α-IL-6/IFNAR1 Ab)
			bin 8: $P = 0.776$ (Cont. vs CXCL12), $P = 0.970$ (CXCL12 vs CXCL12, α-IL-6 Ab), $P = 0.615$ (CXCL12 vs CXCL12, α-IFNAR1 Ab), $P = 0.582$ (CXCL12 vs CXCL12, α-IL-6/IFNAR1 Ab)

Supplementary Table 1 The exact *P*-values in Fig. 4g, l and 10c.
The exact *P*-values, which could not be descripted in each figure legend, are summarized in this table.

Supplementary Table 2

Gene	Class
Bcl11b	Strong layer 5, weak layer 6 and subplate marker (1)
Btg1	Layer 2/3 marker (2)
Cdh13	CSMN general identity (layer 5) (1)
Cited2	CPNs in the deepest part of layer 2/3 (2)
Ctgf	Subplate marker (3)
Cux1	Layer 2/3 and 4 marker (4)
Cux2	Layer 2/3 and 4 marker (4, 5)
Dkk3	Strong layer 6, weak layer 5 marker (1, 6, *)
Epha3	CPNs in the most superficial part of layer 2/3 (2)
Etv1	Layer 5 marker (7)
Fezf2	Strong layer 5, weak layer 6 marker (2, 8)
Foxo1	Layer 5 marker (8)
Foxp2	Layer 6 marker (9, 10)
Frmd4b	CPNs in layer 2/3 (2)
Gnaz	Subplate marker (7)
Grb14	CSMN early development (layer 5) (1)
Hs3st4	Strong layer 6, weak layer 5 marker (6)
Inhba	Layer 2/3 (2)
Kitl	Layer 5 and 6 marker at E18.5 (6)
Ldb2	CSMN early development (layer 5) (1)
Limch1	Layer 2/3 (2)
Lmo3	Layer 5, 6 and subplate marker (11, 12)
Lmo4	Layer 2/3, 4 and 6 marker (1, 13, 14)
Lpl	Layer 2/3 and 5 marker (2)
Lxn	Layer 5 and 6 marker (*)
Mdga1	Layer 2/3 and SVZ at E16.5 (15)
Nr4a2	Subplate marker (16)
Nr4a3	Deep layer marker at E18.5 (*)
Ntng1	Subplate marker (17)
Oma1	CSMN general identity (layer 5) (1)
Pcdh8	Layer 2/3 and 5 marker (6)
Pcp4	Layer 5 and subplate marker (18, *)
Plxnd1	Layer 2/3 and 5 marker (2)
Pou3f1	Strong layer 5, weak layer 2/3 marker (19, 20)
Pou3f2	Layer 2/3 marker (19)
Ppp1r1b	Layer 6 marker (*)
Ptn	CPNs located in the deepest part of layer 2/3 (1, 2)
Rorb	Layer 4 marker (21)
S100a10	Layer 5 marker at E18.5 (*)
Satb2	Layer 2/3 and 4 marker (6, 22)
Sla	Layer 5 and 6 marker (11)
Slitrk1	Layer 2/3, 5, 6 and subplate marker (23, *)
Sox5	Strong layer 6 and subplate, weak layer 5 marker (24)
Syt9	CSMN early development (layer 5) (1)
Tbr1	Layer 6 and subplate marker (25)
Tle4	Strong layer 6, weak layer 5 marker (8)

References

1 Arlotta, P. et al. *Neuron* **45,** 207–221 (2005).
2 Molyneaux, B. J. et al. *J. Neurosci.* **29,** 12343–12354 (2009).
3 Tiong, S. Y. K. et al. *Front Neuroanat.* **13,** 39 (2019).
4 Nieto, M. et al. *J. Comp. Neurol.* **479,** 168–180 (2004).
5 Zimmer, C. et al. *Cereb. Cortex* **14,** 1408–1420 (2004).
6 Alcamo, E. A. et al. *Neuron* **57,** 364–377 (2008).
7 Hevner, R. F. et al. *Dev. Neurosci.* **25,** 139–151 (2003).
8 Chen, B. et al. *Proc. Natl. Acad. SCI. U. S. A.* **102,** 17184–17189 (2005).
9 Ferland, R. J. et al. *J. Comp. Neurol.* **460,** 266–279 (2003).
10 Kast, R. J. et al. *Elife* e42012 (2019).
11 Loo, L. et al. *Nat. Commun.* **10,** 134 (2019).
12 Abellán, A. et al. *Front Neuroanat.* **8,** 59 (2014).
13 Bulchand, S. et al. *Dev. Dyn.* **226,** 460–469 (2003).
14 Harb, K. et al. *Elife* 5, e09531 (2016).
15 Perez-Garcia, C. G. and O'Leary, D. D. M. *Cell Rep.* **14,** 560–571 (2016).
16 Magnani, D. et al. *Cereb. Cortex* **23,** 2542–2551 (2013).
17 Nakashiba, T. et al. *Mech. Dev.* **111,** 47–60 (2002).
18 Hoerder-Suabedissen, A. et al. *Proc. Natl. Acad. SCI. U. S. A.* **110,** 3555–3560 (2013).
19 McEvilly, R. J. et al. *Science* **295,** 1528–1532 (2002).
20 Frantz, G. D. et al. *J. Neurosci.* **14,** 472–485 (1994).
21 Schaeren-Wiemers, N. et al. *Eur. J. Neurosci.* **9,** 2687–2701 (1997).
22 Britanova, O. et al. *Neuron* **57,** 378–392 (2008).
23 Stillman, A. A. et al. *J. Comp. Neurol.* **513,** 21–37 (2009).
24 Kwan, K. Y. et al. *Proc. Natl. Acad. SCI. U. S. A.* **105,** 16021–16026 (2008).
25 Hevner, R. F. et al. *Neuron* **29,** 353–366 (2001).
* Personal observation referring to Allen Brain Atlas (https://portal.brain-map.org)

Supplementary Table 2 The list of all references that we refer to about each cell type- or layer-specific marker in the embryonic brain

Abbreviations: CSMN, corticospinal motor neurons. CPN, callosal projection neurons. CSMNs are reported to be located primarily in cortical L5[26,27]. In rodents and in primates, CPNs, which connect through the corpus callosum, exist across multiple layers and they express not only some common callosal genes, such as Satb2, but also express layer specific genes.

Supplementary Table 3

Direction	*P*-value	Log *P*-value	GO terms	nGenes	Gene symbols
Up-regulated	7.1E-37	36.15	Response to stress	126	*Ifitm3, Cxcl1, Oasl2, Ltbr, Ifit1, Cxcl10, Ccl7, Ccl2, Bst2, Irgm1, Iigp1, Ifit3, Rsad2, Il6, Irf7, Stat1, Lcn2, Zc3hav1, Isg15, Socs1, Stat2, Usp18, Igf2, H2-T23*
Up-regulated	2.61E-34	33.58	Defense response	81	*Ifitm3, Cxcl1, Oasl2, Ifit1, Cxcl10, Ccl7, Ccl2, Bst2, Irgm1, Iigp1, Ifit3, Rsad2, Irf7, Il6, Stat1, Lcn2, Zc3hav1, Isg15, Socs1, Stat2, Usp18, Igf2, H2-T23*
Up-regulated	2.61E-34	33.58	Immune system process	100	*Irf9, Ifitm3, Irf7, Cxcl1, Oasl2, Ltbr, Ifit1, Cxcl10, Ccl7, Ccl2, Bst2, H2-T23, Ifit3, Rsad2, Il6, Irgm1, Stat1, Lcn2, Zc3hav1, Isg15, Socs1, Stat2, Iigp1*
Up-regulated	1.11E-30	29.96	Immune response	75	*Ifitm3, Cxcl1, Cxcl10, Ccl7, Ccl2, Bst2, H2-T23, Il6, Irgm1, Rsad2, Irf7, Stat1, Lcn2, Oasl2, Zc3hav1, Ltbr, Ifit1, Isg15, Socs1, Stat2, Iigp1, Ifit3*
Up-regulated	7.26E-29	28.14	Response to external biotic stimulus	63	*Ifitm3, Cxcl1, Oasl2, Ifit1, Cxcl10, Oasl1, Bst2, Ifit3, Rsad2, Il6, Lcn2, Ccl2, Iigp1, Irf7, Stat1, Zc3hav1, Isg15, Stat2, Usp18, Irgm1, H2-T23*
Up-regulated	4.91E-28	27.31	Response to cytokine	60	*Ifitm3, Cxcl1, Cxcl10, Ccl7, Ccl2, Irgm1, Iigp1, Il6, Ifit1, Bst2, Ifit3, Osmr, Stat1, Lcn2, Isg15, Stat2, Irf7*
Up-regulated	1.83E-27	26.74	Response to external stimulus	92	*Ifitm3, Cxcl1, Oasl2, Ltbr, Ifit1, Cxcl10, Ccl7, Ccl2, Bst2, Ifit3, Rsad2, Il6, Lcn2, Irf7, Stat1, Zc3hav1, Isg15, Stat2, Usp18, Irgm1, H2-T23*
Up-regulated	5.32E-27	26.27	Response to organic substance	106	*Ifitm3, Cxcl1, Cxcl10, Ccl7, Ccl2, Socs1, Irgm1, Iigp1, Il6, Zc3hav1, Ifit1, Bst2, Ifit3, Osmr, Stat1, Lcn2, Isg15, Stat2, Usp18, Irf7*
Up-regulated	1.54E-25	24.81	Innate immune response	51	*Ifitm3, Ccl7, Ccl2, Bst2, Irgm1, Rsad2, Irf7, Stat1, Lcn2, Oasl2, Zc3hav1, Ifit1, Isg15, Socs1, Stat2, Iigp1, Ifit3, H2-T23*
Up-regulated	5.47E-24	23.26	Cellular response to organic substance	86	*Ifitm3, Cxcl1, Cxcl10, Ccl7, Ccl2, Socs1, Irgm1, Iigp1, Il6, Zc3hav1, Ifit1, Ifit3, Osmr, Stat1, Lcn2, Stat2, Irf7*
Up-regulated	8.89E-24	23.05	Immune effector process	49	*Ifitm3, Oasl2, Ifit1, Bst2, H2-T23, Ifit3, Rsad2, Il6, Cxcl1, Cxcl10, Irf7, Stat1, Zc3hav1, Isg15, Stat2, Ccl2, Igf2*
Up-regulated	2.26E-23	22.65	Regulation of multicellular organismal process	98	*Il6, Cxcl1, Zc3hav1, Irf7, Stat1, Cxcl10, Ccl2, Isg15, Socs1, Rsad2, Lcn2, Igf2, H2-T23, Bst2*
Up-regulated	4.71E-23	22.33	Positive regulation of response to stimulus	81	*Ltbr, Ccl7, Ccl2, H2-T23, Il6, Cxcl1, Cxcl10, Rsad2, Irf7, Socs1, Irgm1, Zc3hav1*
Down-regulated	2.83E-06	5.55	Anterograde trans-synaptic signaling	20	*Calb2, Grm3, Grm2, Gabrd, Gabrb1, Gabra3, Gabra5, Slc17a7, Prkcb, Sv2b, Adcy1, Npas4, Egr1, Rapgef4*
Down-regulated	2.83E-06	5.55	Cell-cell signaling	29	*Calb2, Grm3, Grm2, Gabrd, Gabrb1, Gabra3, Gabra5, Slc17a7, Rapgef4, Prkcb, Spp1, Sv2b, Lhx5, Adcy1, Egr1, Npas4*
Down-regulated	2.83E-06	5.55	Establishment of localization	57	*Caly, Gabrd, Gabrb1, Gabra3, Fxyd7, Gabra5, Slc17a7, Slc1a2, Spon2, Rapgef4, Prkcb, Hdac9, Grm2, Kcnip2, Tmem163, Astn2, Spp1, Cxcr4, Sv2b, Adcy1*
Down-regulated	6.73E-06	5.17	Transport	55	*Caly, Gabrd, Gabrb1, Gabra3, Fxyd7, Gabra5, Slc17a7, Slc1a2, Spon2, Rapgef4, Prkcb, Hdac9, Grm2, Kcnip2, Tmem163, Astn2, Spp1, Cxcr4, Sv2b, Adcy1*
Down-regulated	7.71E-06	5.11	Cell differentiation	53	*Epha10, Spp1, Bmp3, Lhx5, Brinp3, Sox21, Fos, Rai14, Kcnip2, Gabrb1, Cxcr4, Npas4, Ndnf, Fabp7, Adcy1, Shox2, Rxfp1, Egr1, Gabra5, Rapgef4, Ctsl*
Down-regulated	7.71E-06	5.11	Regulation of localization	41	*Sst, Fxyd7, Astn2, Rapgef4, Prkcb, Hdac9, Kcnip2, Caly, Spp1, Cxcr4, Slc17a7, Slc1a2, Adcy1, Egr1*
Down-regulated	1.83E-05	4.74	Regulation of transport	32	*Fxyd7, Rapgef4, Prkcb, Hdac9, Kcnip2, Caly, Spp1, Cxcr4, Slc17a7, Slc1a2, Adcy1*
Down-regulated	2.02E-05	4.69	Regulation of cell communication	45	*Calb2, Grm3, Grm2, Igfbp5, Bmp3, Rcan2, Dhrs3, Prkcb, Sst, Spp1, Blnk, Adcy1, Shox2, Egr1, Npas4, Rapgef4*
Down-regulated	2.06E-05	4.69	Ion transport	29	*Gabrd, Gabrb1, Gabra3, Fxyd7, Gabra5, Slc1a2, Prkcb, Slc17a7, Grm2, Kcnip2, Tmem163, Cxcr4*
Down-regulated	2.06E-05	4.69	Regulation of response to stimulus	49	*Bmp3, Rcan2, Dhrs3, Spon2, Sst, Spp1, Cxcr4, Blnk, Fabp7, Shox2, Egr1, Prkcb*

Supplementary Table 3 GO analysis of upregulated or downregulated genes
The list shows symbol genes which are categorized to each GO term group.

Supplementary References

1. Arlotta, P. et al. Neuronal subtype-specific genes that control corticospinal motor neuron development in vivo. *Neuron* **45,** 207–221 (2005).
2. Molyneaux, B. J. et al. Novel subtype-specific genes identify distinct subpopulations of callosal projection neurons. *J. Neurosci.* **29,** 12343–12354 (2009).
3. Tiong, S. Y. K. et al. Kcnab1 Is Expressed in Subplate Neurons With Unilateral Long-Range Inter-Areal Projections. *Front Neuroanat.* **13,** 39 (2019).
4. Nieto, M. et al. Expression of Cux-1 and Cux-2 in the subventricular zone and upper layers II-IV of the cerebral cortex. *J. Comp. Neurol.* **479,** 168–180 (2004).
5. Zimmer, C. et al. Dynamics of Cux2 expression suggests that an early pool of SVZ precursors is fated to become upper cortical layer neurons. *Cereb. Cortex* **14,** 1408–1420 (2004).
6. Alcamo, E. A. et al. Satb2 regulates callosal projection neuron identity in the developing cerebral cortex. *Neuron* **57,** 364–377 (2008).
7. Hevner, R. F. et al. Beyond laminar fate: toward a molecular classification of cortical projection/pyramidal neurons. *Dev. Neurosci.* **25,** 139–151 (2003).
8. Chen, B. et al. Fezl regulates the differentiation and axon targeting of layer 5 subcortical projection neurons in cerebral cortex. *Proc. Natl. Acad. SCI. U. S. A.* **102,** 17184–17189 (2005).
9. Ferland, R. J. et al. Characterization of Foxp2 and Foxp1 mRNA and protein in the developing and mature brain. *J. Comp. Neurol.* **460,** 266–279 (2003).
10. Kast, R. J. et al. FOXP2 exhibits projection neuron class specific expression, but is not required for multiple aspects of cortical histogenesis. *Elife* **8,** e42012 (2019).
11. Loo, L. et al. Single-cell transcriptomic analysis of mouse neocortical development. *Nat. Commun.* **10,** 134 (2019).
12. Abellán, A. et al. Combinatorial expression of Lef1, Lhx2, Lhx5, Lhx9, Lmo3, Lmo4, and Prox1 helps to identify comparable subdivisions in the developing hippocampal formation of mouse and chicken. *Front Neuroanat.* **8,** 59 (2014).
13. Bulchand, S. et al. Dynamic spatiotemporal expression of LIM genes and cofactors in the embryonic and postnatal cerebral cortex. *Dev. Dyn.* **226,** 460–469 (2003).
14. Harb, K. et al. Area-specific development of distinct projection neuron subclasses is regulated by postnatal epigenetic modifications. *Elife* **5,** e09531 (2016).
15. Perez-Garcia, C. G. and O'Leary, D. D. M. Formation of the Cortical Subventricular Zone Requires MDGA1-Mediated Aggregation of Basal Progenitors. *Cell Rep.* **14,** 560–571 (2016).
16. Magnani, D. et al. Gli3 controls subplate formation and growth of cortical axons. *Cereb. Cortex* **23,** 2542–2551 (2013).
17. Nakashiba, T. et al. Complementary expression and neurite outgrowth activity of netrin-G subfamily members. *Mech. Dev.* **111,** 47–60 (2002).

18. Hoerder-Suabedissen, A. et al. Expression profiling of mouse subplate reveals a dynamic gene network and disease association with autism and schizophrenia. *Proc. Natl. Acad. SCI. U. S. A.* **110,** 3555–3560 (2013).
19. McEvilly, R. J. et al. Transcriptional regulation of cortical neuron migration by POU domain factors. *Science* **295,** 1528–1532 (2002).
20. Frantz, G. D. et al. Regulation of the POU domain gene SCIP during cerebral cortical development. *J. Neurosci.* **14,** 472–485 (1994).
21. Schaeren-Wiemers, N. et al. The expression pattern of the orphan nuclear receptor RORbeta in the developing and adult rat nervous system suggests a role in the processing of sensory information and in circadian rhythm. *Eur. J. Neurosci.* **9,** 2687–2701 (1997).
22. Britanova, O. et al. Satb2 is a postmitotic determinant for upper-layer neuron specification in the neocortex. *Neuron* **57,** 378–392 (2008).
23. Stillman, A. A. et al. Developmentally regulated and evolutionarily conserved expression of SLITRK1 in brain circuits implicated in Tourette syndrome. *J. Comp. Neurol.* **513,** 21–37 (2009).
24. Kwan, K. Y. et al. SOX5 postmitotically regulates migration, postmigratory differentiation, and projections of subplate and deep-layer neocortical neurons. *Proc. Natl. Acad. SCI. U. S. A.* **105,** 16021–16026 (2008).
25. Hevner, R. F. et al. Tbr1 regulates differentiation of the preplate and layer 6. *Neuron* **29,** 353–366 (2001).
26. Lodato, S and Arlotta, P. Generating neuronal diversity in the mammalian cerebral cortex. *Annu. Rev. Cell Dev. Biol.* **31,** 699–720 (2015).
27. Fame, R. M. et al. Development, specification, and diversity of callosal projection neurons. *Trends. Neurosci.* **34,** 41–50 (2011).
