# Supplementary material for: Transient microglial absence assists postmigratory cortical neurons in proper differentiation
Source: Nat Commun. 2020 Apr 2;11:1631. doi: 10.1038/s41467-020-15409-3 (PMC7118101; doi:10.1038/s41467-020-15409-3)
Supplement: Supplementary file 3 — Description of Additional Supplementary Information [file 41467_2020_15409_MOESM3_ESM.pdf]

**Description of Additional Supplementary Files**

File Name: **Supplementary Data 1**

Description: **1 FPKM values for neuronal subtype-associated genes in RNA-Seq analysis** The individual FPKM values for each sample (*n* = 3) in the neurons$^{Cont}$ and neurons$^{MG}$ groups are shown. The *p*-value was evaluated by Welch's T test. The *q*-value (FDR) was calculated from the two-tailed *p* values using the R software package qvalue.

File Name: **Supplementary Movie 1**

Description: **Live imaging of microglia initially positioned in the CP**

Live imaging of microglia in cortical slices derived from E14 CX3CR1-GFP mouse. Microglia initially positioned in the CP tend to migrate towards the meninges. Time-lapse imaging covers a period of 8 hr (one image every 5 min). Yellow arrowhead shows the soma of microglia. White broken lines show the meninges. Scale bar, 50 µm.

File Name: **Supplementary Movie 2**

Description: **Live imaging of microglia the meninges-detached cortical slice**

Live-imaging of microglia in the meninges-detached cortical slice derived from E14 CX3CR1-GFP mouse. The basalward-predominant migration of microglia in the CP was abolished. Time-lapse imaging covers a period of 8 hr (one image every 5 min). Pink arrowhead shows the soma of microglia migrated apicalward and cyan arrowhead shows that of stationary microglia. Scale bar, 50 µm.

File Name: **Supplementary Movie 3**

Description: ***In utero* observation of microglia in the SVZ**

*In utero* observation using two-photon microscopy of microglia at the depth of 250 µm from the cranial surface (corresponding to the SVZ) of E15 CX3CR1-GFP mouse. Microglial movement was monitored for 90 min (1 image per 30 sec). Scale bar, 30 µm.

File Name: **Supplementary Movie 4**

Description: ***In utero* observation of microglia in the CP**

*In utero* observation using two-photon microscopy of microglia in the CP of E14 CX3CR1-GFP mouse. Image sequences covered the whole CP region were captured in 2-µm pitch and were converted to Z-stack image. Microglial movement was monitored for 90 min (1 image/ 5 min). Scale bar, 50 µm.

File Name: **Supplementary Movie 5**

Description: **Live imaging of microglia initially positioned in the inner region**

Live-imaging of microglia in cortical slices derived from E14 CX3CR1-GFP mouse. Microglia initially positioned in the inner region (i.e., IZ/SVZ) tended to migrate apicalward. Time-lapse imaging covers a period of 8 hr (one image every 5 min). Pink arrowheads show the somata of microglia. Scale bar, 50 µm.

File Name: **Supplementary Movie 6**

Description: **Comparison of microglial movements in the CP between WT and *Cxcr4*$^{-/-}$ mice**

Time-lapse imaging of microglia positioned in the CP of cortical slices, comparing E14 WT and *Cxcr4*$^{-/-}$ mice, both of which have the *Cx3cr1*$^{+/-}$ background. The movie covers a period of 8 hr (one image every 5 min). Arrowheads indicate the somata of microglia. Scale bar, 50 µm.

File Name: **Supplementary Movie 7**

Description: **Comparison of microglial movements in the SVZ/IZ between WT and *Cxcr4*$^{-/-}$ mice**

Time-lapse imaging of microglia positioned in the inner region (SVZ/IZ), comparing between E14 WT and *Cxcr4*$^{-/-}$ cerebral wall slices. It covers a period of 8 hr (one image every 5 min). Arrowheads indicate the somata of microglia. Scale bar, 50 µm.

File Name: **Supplementary Movie 8**
Description: **Live imaging of microglia in slices decorated with CXCL12-soaked beads**
Live-imaging of microglia in E15 CX3CR1-GFP mouse cortical slices, the basal surfaces of which were decorated with CXCL12-soaked beads. The movie covers a period of 8 hr (one image every 10 min). White broken lines show the outline of the cerebral slice and the border between the CP and IZ. Yellow arrowheads at the 3-hr time point indicate microglia that were recruited to the CP from the SVZ/IZ. Scale bar, 100 µm.

File Name: **Supplementary Movie 9**
Description: **Live imaging of transplanted microglia in the CP of WT mouse cortical slices**
Live-imaging of transplanted CX3CR1-GFP$^{+}$ microglia in E15 ICR mouse cortical slices. White broken lines indicate the CP. It covers a period of 8 hr (one image every 10 min). Scale bar, 50 µm.
