# Supplementary material for: Transient microglial absence assists postmigratory cortical neurons in proper differentiation
Source: Nat Commun. 2020 Apr 2;11:1631. doi: 10.1038/s41467-020-15409-3 (PMC7118101; doi:10.1038/s41467-020-15409-3)
Supplement: Supplementary file 14 — Reporting Summary [file 41467_2020_15409_MOESM14_ESM.pdf]

nature research

Corresponding author(s): Yuki Hattori and Takaki Miyata

Last updated by author(s): Feb 27, 2020

Reporting Summary

Nature Research wishes to improve the reproducibility of the work that we publish. This form provides structure for consistency and transparency in reporting. For further information on Nature Research policies, see Authors & Referees and the Editorial Policy Checklist .

Statistics

For all statistical analyses, confirm that the following items are present in the figure legend, table legend, main text, or Methods section.

n/a	Confirmed	
☐	☒	The exact sample size (*n*) for each experimental group/condition, given as a discrete number and unit of measurement
☐	☒	A statement on whether measurements were taken from distinct samples or whether the same sample was measured repeatedly
☐	☒	The statistical test(s) used AND whether they are one- or two-sided<br>*Only common tests should be described solely by name; describe more complex techniques in the Methods section.*
☐	☒	A description of all covariates tested
☐	☒	A description of any assumptions or corrections, such as tests of normality and adjustment for multiple comparisons
☐	☒	A full description of the statistical parameters including central tendency (e.g. means) or other basic estimates (e.g. regression coefficient) AND variation (e.g. standard deviation) or associated estimates of uncertainty (e.g. confidence intervals)
☐	☒	For null hypothesis testing, the test statistic (e.g. *F*, *t*, *r*) with confidence intervals, effect sizes, degrees of freedom and *P* value noted<br>*Give P values as exact values whenever suitable.*
☐	☒	For Bayesian analysis, information on the choice of priors and Markov chain Monte Carlo settings
☐	☒	For hierarchical and complex designs, identification of the appropriate level for tests and full reporting of outcomes
☐	☒	Estimates of effect sizes (e.g. Cohen's *d*, Pearson's *r*), indicating how they were calculated

Our web collection on statistics for biologists contains articles on many of the points above.

Software and code

Policy information about availability of computer code

Data collection	Image data were collected using FV10-ASW software version 4.1 on Fluoview FV1000 (Olympus) and NIS-Elements software AR Analysis version 5.01.00 on TiEA1R (Nikon) and A1Rsi (Nikon). Live-imaging data were collected using CV1000 software version 1.06.06 on CellVoyager CV1000 (Yokogawa Electric Corporation). Flow cytometry analysis was performed using FACSDiva software version 8.0 on FACS Canto II and FACS SORP Aria II (BD Biosciences). RNA samples were sequenced on the Illumina NovaSeq6000 platform using a 100 bp paired-end strategy. ELISA data were obtained using PowerScan4.
Data analysis	For RNA-Seq, read qualities were assessed by the FASTQC tool on Galaxy. Reads were mapped to the mouse genome assembly (mm10) using TopHat version 2.1.1 using the corresponding sample's mean inner distance between mate pairs. The mRNA read counts were quantified during transcript assembly with Cufflinks version 2.2.1.2. For the heat map, the count data were transformed using the DESeq2 algorithm. Seeking an unbiased approach to pathway analysis, we used the gene set enrichment analysis (GSEA) tool developed by Broad Institute (http://software.broadinstitute.org/gsea/index.jsp), which identifies groups of coordinately regulated genes present in gene sets annotated in the Molecular Signatures Database (MSigDB). Flow cytometry data were analyzed using FlowJo software version 7.6. Statistical analyses were performed using R software version 3.6.0.

For manuscripts utilizing custom algorithms or software that are central to the research but not yet described in published literature, software must be made available to editors/reviewers. We strongly encourage code deposition in a community repository (e.g. GitHub). See the Nature Research guidelines for submitting code & software for further information.

Data

Policy information about availability of data

All manuscripts must include a data availability statement. This statement should provide the following information, where applicable:

- Accession codes, unique identifiers, or web links for publicly available datasets
- A list of figures that have associated raw data
- A description of any restrictions on data availability

The source data for all experiments in this study are provided as a Source Data file. The raw data have been deposited in the DNA Data Bank of Japan (DDBJ) under

the DRX accession number: DRX199371–DRX199376. These sequence data are also available at NCBI Sequence Read Archive (SRA) under the ID code: DRP005827 (https://trace.ncbi.nlm.nih.gov/Traces/sra/?study=DRP005827).

Field-specific reporting

Please select the one below that is the best fit for your research. If you are not sure, read the appropriate sections before making your selection.

- [x] Life sciences
- [ ] Behavioural & social sciences
- [ ] Ecological, evolutionary & environmental sciences

For a reference copy of the document with all sections, see nature.com/documents/nr-reporting-summary-flat.pdf

Life sciences study design

All studies must disclose on these points even when the disclosure is negative.

Sample size	No statistical methods were used to predetermine the sample size owing to experimental limitations. Sample size was determined to be adequate based on the magnitude and consistency of measurable differences between groups.
Data exclusions	No samples were basically excluded from the analysis. We only excluded the data obtained from failed experiments by some reasons, e.g., failure in sample preparations.
Replication	We confirmed that replicate experiments were successful by repeating at least three times for all experiments.
Randomization	No randomization of mice was performed. Mice analyzed were litter mates whenever possible.
Blinding	Basically, investigators were blinded during experiments.

Reporting for specific materials, systems and methods

We require information from authors about some types of materials, experimental systems and methods used in many studies. Here, indicate whether each material, system or method listed is relevant to your study. If you are not sure if a list item applies to your research, read the appropriate section before selecting a response.

Materials & experimental systems

n/a	Involved in the study	
☐	☒	Antibodies
☐	☒	Eukaryotic cell lines
☒	☐	Palaeontology
☐	☒	Animals and other organisms
☒	☐	Human research participants
☒	☐	Clinical data

Methods

n/a	Involved in the study	
☒	☐	ChIP-seq
☐	☒	Flow cytometry
☒	☐	MRI-based neuroimaging

Antibodies

Antibodies used	Information on all antibodies used in the study (their supplier name, catalog number and the concentration) was described in the Method section.
Validation	Validation about all antibodies was described on the manufacturer's website. For some antibodies (such as anti-Satb2 antibody, anti-Ctip2 antibody, anti-Brn2 and anti-RORb antibody) that we used for intracellular FACS staining, we separately evaluated whether these antibodies could be used because the information about the application for FACS was missed in the manufacturer's site. The antibody concentration we determined is provided in the Method section.

Eukaryotic cell lines

Policy information about cell lines

Cell line source(s)	NB2a mouse neuroblastoma cell line was introduced from RIKEN BRC.
Authentication	Short Tandem Repeat (STR) profiling was applied.
Mycoplasma contamination	NB2a tested negative for mycoplasma.
Commonly misidentified lines (See ICLAC register)	No use.

Animals and other organisms

Policy information about studies involving animals; ARRIVE guidelines recommended for reporting animal research

Laboratory animals	CX3CR1-GFP transgenic (Tg) male mice (8–24 weeks) were mated with ICR female mice (8–24 weeks), thereby we obtained the CX3CR1-GFP heterozygous embryos (E12–E18). We used both these male and female mice in this study. For Cxcr4 knock out mice, Gadd45g-d4Venus Tg mice and ICR mice, we used both of male and female embryos (E12–E18). Resources for these mice were listed in Method section. All mice were maintained under specific pathogen-free conditions, and were housed at 22–24°C temperature with 40-60% humidity at Nagoya University. A 12-hour light/12-hour dark cycle was used.
Wild animals	No wild animals were used in this study.
Field-collected samples	No use.
Ethics oversight	The animal experiments were conducted according to Japanese Act on Welfare and Management of Animals, Guidelines for Proper Conduct of Animal Experiments (published by Science Council of Japan), Fundamental Guidelines for Proper Conduct of Animal Experiment and Related Activities in Academic Research Institutions (published by Ministry of Education, Culture, Sports, Science and Technology, Japan). All protocols for animal experiments were approved by the Institutional Animal Care and Use Committee of Nagoya University (No. 29006).

Note that full information on the approval of the study protocol must also be provided in the manuscript.

Flow Cytometry

Plots

Confirm that:

- [x] The axis labels state the marker and fluorochrome used (e.g. CD4-FITC).
- [x] The axis scales are clearly visible. Include numbers along axes only for bottom left plot of group (a 'group' is an analysis of identical markers).
- [x] All plots are contour plots with outliers or pseudocolor plots.
- [x] A numerical value for number of cells or percentage (with statistics) is provided.

Methodology

Sample preparation	Sample preparation was described in online method. Briefly, freshly isolated pallial walls were treated with trypsin (0.05%, 3 min at 37 °C). Dissociated pallial cells were filtered through a 40-µm strainer (Corning, Corning, NY, USA) to eliminate all remaining cell debris and then resuspended in DMEM containing 5% fetal bovine serum (FBS), 5% horse serum (HS) and penicillin/streptomycin (50 U per ml, each).
Instrument	FACS SORP Aria II and FACS Canto II.
Software	FACSDiva Software (ver. 8.0) for data collection and FlowJo software (ver. 7.6) for data analysis.
Cell population abundance	About 10 million cerebral wall cells were sorted to obtain one hundred thousand microglia. About 1 million cerebral wall cells were sorted to obtain half a million Gadd45g-d4Venus+ cells. Purity was assessed by checking GFP fluorescence in a fluorescence microscopy. For intracellular staining, two hundred thousand cells (in vitro-prepared neurons) per sample were prepared.
Gating strategy	For cell-sorting, mouse cerebarl wall cells were gated on a forward scatter (FSC)/side scatter (SSC) plot. Debris and dead cells were exclude, and then CX3CR1-GFP+ or Gadd45g-d4Venus+ cells were further gated and sorted. For intracellular staining, about 5,000 or 3,000 cells (depending on the experiment), which were gated on a FSC/SCC plot for debris exclusion, were analyzed for each sample.

- [x] Tick this box to confirm that a figure exemplifying the gating strategy is provided in the Supplementary Information.
